# Morphology Effects on Free Energies of Proton-Coupled Electron Transfer in Polyoxotungstates

**DOI:** 10.1021/acs.inorgchem.5c02910

**Published:** 2025-10-23

**Authors:** Andreas Towarnicky, Zhou Lu, Ellen M. Matson, Giannis Mpourmpakis

**Affiliations:** 1 Department of Chemical Engineering, 6614 University of Pittsburgh, Pittsburgh, Pennsylvania 15261, United States; 2 Department of Chemistry, 6927 University of Rochester, Rochester, New York 14627, United States; 3 School of Chemical Engineering, National Technical University of Athens (NTUA), Athens GR-15780, Greece

## Abstract

Polyoxotungstates have previously been established to facilitate the hydrogenation of small molecule substrates via hydrogen atom transfer from reactive hydroxyl groups formed at the assembly surface. Understanding structure–function relationships that dictate the thermochemistry and kinetics of proton-coupled electron transfer is key to controlling this chemistry. In this work, we combine comprehensive electrochemical experiments and density functional theory calculations to address how different polyoxotungstate morphologies, specifically W_6_O_19_^–2^, W_10_O_32_^–4^, SiW_12_O_40_^–4^, and P_2_W_18_O_62_^–6^, affect the bond dissociation free energies of surface hydroxides (BDFE­(O–H)) formed upon reduction of the assembly in acidic media. Our results reveal increasing hydroxide bond strengths with increasing cluster size, and that anisotropic cluster geometries result in substantial thermodynamic differentiation of H-binding sites. We demonstrate an excellent agreement between theory and experiments on the reported BDFE­(O–H) values and, importantly, we elucidate how cluster size and shape affect electronic properties (local charges and frontier molecular orbitals), giving rise to sites with increased preference for hydrogen binding, demonstrated in higher BDFE­(O–H). Overall, this work aids the understanding and design of polyoxometalates exhibiting surface sites with tailored interaction strengths.

## Graphical abstract

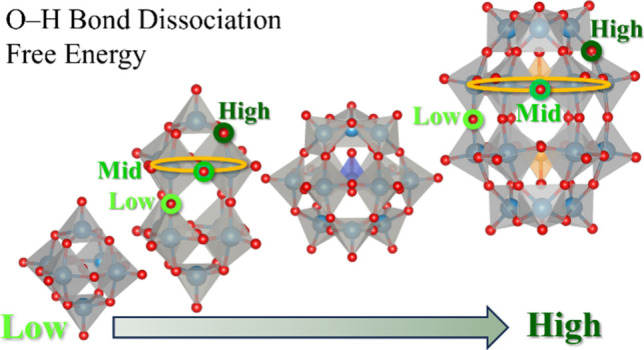

## Introduction

Metal oxides have demonstrated utility in numerous fields,^1^ including catalysis,^2^ and their chemical versatility provides for further lucrative applications.^3,4^ Foundational to their reactive behavior are the dynamics of proton-coupled electron transfer (PCET),^5–7^ which are key to many industrially relevant chemistries, such as O_2_ reduction,^8^ H_2_ evolution,^9^ H_2_O oxidation,^10^ hydrogenation,^11^ and C–H bond activation.^12^ However, improving our understanding of metal oxide reactivity remains challenging due to structural complexities of extended systems, including diversity in composition, morphology and surface coordination. Polyoxometalates (POMs) are connected polyhedra of metal-oxide building blocks,^13,14^ with a wide range of applications.^15–21^ Importantly, POMs are atomically precise and well-suited to both experimental and computational investigation.^22^ Additionally, POMs possess similar structural features as extended systems, enabling the study of metal oxides via molecular moieties.^23,24^

Of particular interest is to understand the structure–function relationships of metal oxides that dictate PCET thermochemistry. Focusing on tungsten trioxide nanomaterials and polyoxotungstates (POTs), previous works by our research team have shown how varying hydrogen intercalation affects the acid–base properties of tungsten oxides, altering PCET thermodynamics,^9,25,26^ and that relatively weak surface O–H bonds of Keggin-type POTs may effect selective hydrogenations.^27^ An essential thermochemical descriptor of PCET activity is the bond dissociation free energy (BDFE),^28^ providing fungible and unambiguous bond characterization,^29,30^ helping describe *e* ^*–*^ and H^+^ activities,^31^ reaction directions,^32^ approximate reaction rates,^24^ and thermal reactivities,^28^ directly applicable to the BDFE­(O–H) of POM surface hydroxide bonds.^22^

By virtue of their building-*block*-like assembly, POTs may be synthesized in a variety of sizes and shapes (Figure 1),^13^ and it is desirable to understand how these aspects may influence their PCET behavior, which may tailor their application performance.^33,34^ Prominent POT morphologies include the octahedral Lindqvist-type^35^ [W_6_O_19_] and cuboctahedral Keggin-type^36^ α-[XW_12_O_40_] clusters. The Keggin cluster is larger than the Lindqvist in size, with twice the number of tungsten centers, and both are structurally isotropic. Previous investigation of the acid-dependent redox properties of the Lindqvist-type polyoxoanion, **W** **_6_** **O** **_19_** **^–2^**, revealed no change in the reduction potential of this tungsten oxide cluster across solution p*K*_a_ from 39.5 to 0.7, suggesting that the surface of the assembly is too acidic to form stable surface hydroxyl groups under the examined experimental conditions.^37^ However, similar studies of the phosphate-centered Keggin anion (**PW** **_12_** **O** **_40_** **^–3^**) revealed that the assembly forms weak surface O–H bonds (BDFE­(O–H) ∼ 48 kcal/mol^27^) upon reduction in the presence of acid, below the comparable molecular hydrogen BDFE­(H–H) = 52.0 kcal/mol per H atom in acetonitrile.^29,38^ In this work we expand our investigations to include two additional POT morphologies, the decatungstate^39^ [W_10_O_32_] and Wells-Dawson-type^40–42^ α-[X_2_W_18_O_62_] clusters, which may be considered as the fusion of two cap-removed Lindqvist clusters and two cap-removed Keggin clusters, respectively. It is known that increasing cluster size decreases the energy gap between highest occupied molecular orbital (HOMO) and lowest unoccupied molecular orbital (LUMO),^43^ thus one may expect BDFE­(O–H) to differ with cluster size, however, this does not address shape effects, and the extent and regularity of potential BDFE­(O–H) changes with different morphologies is unclear. For example, the **W** **_10_** **O** **_32_** **^–4^** cluster is smaller than **PW** **_12_** **O** **_40_** **^–3^**, but serves as an especially efficient photocatalyst for hydrogen atom transfer and activator of refractory organic substrates.^44–46^ By considering these cluster types we may investigate size effects from just 6 W centers to three times as large, and of changing shape from the approximately spherical Lindqvist and Keggin clusters to the approximately 2:1 ellipsoidal decatungstate and Wells-Dawson.

**Figure 1:**
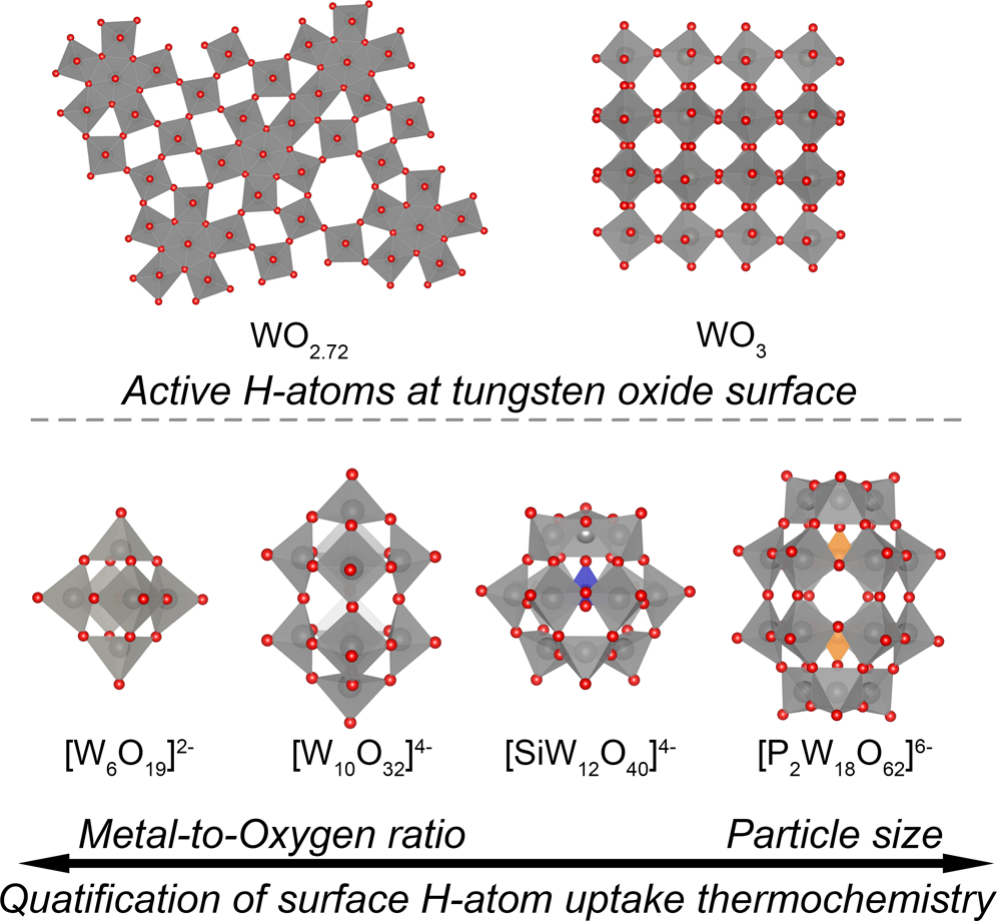
Overview of H atom uptake chemistry on tungsten oxide materials (top) and polyoxotungstate clusters studied in this work (bottom).

There has also been disagreement on the preferred H-binding sites of the clusters, which are key to understanding how geometric effects give rise to changes in BDFE­(O–H). Prior work has explored favored protonation sites of the decatungstate,^47^ Keggin,^48^ as well as of the Lindqvist and Wells-Dawson^49^ clusters; but consistency, common understanding across clusters, and corroborating experimental and theoretical results with complete PCET thermodynamics have been elusive. For example, X-ray diffraction work by Sasaki et. al in the late 1980s inferred that photoreduced **W** **_10_** **O** **_32_** **^–4^** may interact with protons through ion pairing interactions,^50^ as opposed to forming discrete surface hydroxide bonds. In such a case, PCET behavior could be expected to be drastically different than that of clusters with covalent hydroxide bonds. However, diisopropylammonium counterions in their crystal structures formed N–H---O hydrogen bonds,^50^ suggesting possible accompanying affinity for protons, and notably the N–H---O hydrogen bonds formed with the bridging oxygen (O_B_) on the ends of the cluster, rather than equatorial bridging positions, or any terminal (O_T_) oxygens.^50^ More recent theoretical work by Ravelli et al. on **W** **_10_** **O** **_32_** **^–4^** found that O_B_ on the cluster polar ends were similarly thermodynamically favored as equatorial O_T_, with the latter preferred by 0.53 kcal/mol.^47^ Subsequent work by Waele et al. suggested an O_T_ on the end of **W** **_10_** **O** **_32_** **^–4^** was preferred for hydrogen atom transfer.^44^ Theoretical work by Bridgeman and Cavigliasso found minor structural changes upon **W** **_10_** **O** **_32_** **^–4^** reduction by hydrogen atoms,^51^ offering an alternative understanding from the work of Sasaki et al. where a lack of structural changes suggested ionic coordination.^50^ Other recent theoretical work has indicated the end O_B_ as the preferred site for hydrogen atom transfer.^52,53^ Despite this active consideration, BDFE­(O–H) values with experimental confirmation for the reduced and protonated decatungstate **W** **_10_** **O** **_32‑x_** **(OH)** **_*x*_** **^–4^** have not been disclosed. Nor have BDFE­(O–H) been reported for the **W** **_6_** **O** **_19‑x_** **(OH)** **_*x*_** **^–2^**, **SiW** **_12_** **O** **_40‑x_** **(OH)** **_*x*_** **^–4^**, or **P** **_2_** **W** **_18_** **O** **_62‑x_** **(OH)** **_*x*_** **^–6^** clusters. Such gaps in understanding add to confusion within the field over the influences of cluster size, shape, and specific site geometries on BDFE­(O–H), rendering prescriptive formulas for the design of clusters with targeted PCET thermochemistry impracticable.

Herein, we describe the BDFE­(O–H) and *e* ^*–*^/H^+^ activities of a series of POT clusters via comprehensive potential-p*K*_a_ analyses and Density Functional Theory (DFT) calculations. The selected clusters probe size effects, as well as the influence of changing POT length/width ratio (Figure 1). The computational and experimental results are in congruence and provide rational explanations for the changes in the strength of cluster hydroxide bonds. The combined theoretical and experimental observations provide insight into the impacts of cluster size, shape, specific site geometries, and metal oxidation state on the BDFE­(O–H), introducing structure–function relationships on POMs.

## Experimental Methods

All the electrochemistry measurements were carried out in a UniLab MBraun N_2_-filled glovebox; all glassware was oven-dried and cooled in an evacuated antechamber prior to use. Solvents were dried, deoxygenated, and stored over 3 Å molecular sieves that were activated prior to use. [^*n*^Bu_4_N]_4_[W_10_O_32_], [^*n*^Bu_4_N]_4_[SiW_12_O_40_], and [^*n*^Bu_4_N]_6_[P_2_W_18_O_62_] were synthesized according to the literature reports^54,55^ and recrystallized before moving into glovebox.

All cyclic voltammograms (CVs) were collected by using a BioLogic SP-150 potentiostat and acquired with EC-Lab software. Glassy carbon disc (3 mm in diameter, CH Instruments, USA) and a Pt wire were used as working and counter electrode, respectively. A nonaqueous Ag/Ag^+^ reference electrode with 1 mM AgNO_3_ and 100 mM [^*n*^Bu_4_N]­PF_6_ in acetonitrile (BASi, USA) was also used. The scan rate of all CVs was 100 mV/s unless otherwise specified. Each CV was internally calibrated by Fc^+/0^ redox couple. After mapping redox potentials of POT clusters with the p*K*_a_ values of corresponding organic acids, the POT p*K*_a_ uncertainties were estimated by adopting 95% confidence intervals. BDFE­(O–H) values for each POT were calculated using the Bordwell equation:^56^

$$\mathrm{BDFE}(O-H)=1.37pK_{a}+23.06E^{{}^{\circ}}+C_{G}$$ 1

p*K*_a_ and *E*° are experimentally determined and can be derived from the potential-p*K*_a_ diagram and *C*_*G*_ is the conversion term for the *E°*(*H* ^+^/*H* ^•^) couple, equal to 52.6 kcal/mol vs Fc^+/0^ in acetonitrile.^29^

## Computational Methods

DFT calculations were employed using the Gaussian 16 program package.^57^ The hybrid functional B3LYP^58^ was used together with the LANL2DZ basis set.^59^ Implicit solvation was utilized via self-consistent reaction field^60^ with acetonitrile as solvent, corresponding to the experiments (SCRF = (SMD, acetonitrile)). This level of theory resulted in very good agreement in BDFE­(O–H)_avg_ between theory and experiments in polyoxovanadates,^22^ and is computationally tractable while comparably accurate to alternative levels of theory.^61^ Select trends were further evaluated with alternative basis set combination including def2-TZVP for tungsten, with the corresponding effective core potential, and def2-SVP for all other atoms.^62–64^

Cluster geometries and counterion locations were obtained from available experimental crystal structure data.^65–70^ For computational efficiency, tetramethylammonium counterions were used instead of the experimental tetrabutylammonium for clusters **W** **_6_** **O** **_19_** **^–2^**, **W** **_10_** **O** **_32_** **^–4^**, and **P** **_2_** **W** **_18_** **O** **_62_** **^–6^**, and the trimethylammonium counterions in the analogous **PW** **_12_** **O** **_40_** **^–3^** crystal structure were used for **SiW** **_12_** **O** **_40_** **^–4^**. DFT-optimized cluster structures are illustrated in Figure 3 with counterions hidden for clarity, and in Figure S1 with counterion locations displayed. For the H-reduced clusters, when multiple sites were possible for hydrogen atom binding, the hydrogens were placed diametrically per cluster symmetry, avoiding steric hindrance. All distinct oxygen site types were evaluated for 2H reduction. Oxygen site uniqueness was assessed according to prevailing cluster symmetry as if the counterions were not present.

All structures were fully optimized to standard tolerances (RMS force ≤ 3 × 10^–4^ Hartree per Bohr) or even tighter criteria. Vibrational frequencies were computed for the optimized geometries, confirming all minima to be free of any imaginary frequencies. Thermochemical values were determined at 298.15 K and 1 atm, corresponding to experimental conditions. Natural bond orbital (NBO)^71^ analysis was used to determine atomic charges, examine molecular orbitals and plot electron density differences.^72^ Visualizations were developed using GaussView 6 and Vesta 3 software.^73,74^

Gibbs free energy values were utilized to consider the relative energetics of different spin states. Elevated spin states were evaluated for each structure investigated, up to a multiplicity corresponding to (#W^V^ + 2) unpaired electrons, where #W^V^ is the number of reduced tungsten atoms (e.g., **W** **_10_** **O** **_28_** **(OH)** **_4_** **^–4^** with 4 W^V^ centers was investigated up to septet multiplicity). Beyond #W^V^ unpaired electrons, a significant increase in calculated energy was invariably observed. The relevant low-energy results were checked for spin contamination and re-evaluated with rB3LYP if necessary. The lowest energy uncontaminated spin states were utilized for BDFE­(O–H) calculations via eqs 2 and 3 for pertinent comparisons:

$$\mathrm{BDFE}(O-H{)}_{x}^{\mathrm{DFT}}=G_{(x-1)H}^{\mathrm{POT}}-G_{xH}^{\mathrm{POT}}+G^{H^{•}}$$ 2

$$\mathrm{BDFE}(O-H{)}_{x,\mathrm{avg}}^{\mathrm{DFT}}=\frac{1}{2}[G_{(x-2)H}^{\mathrm{POT}}-G_{xH}^{\mathrm{POT}}+2G^{H^{•}}]$$ 3

Herein G stands for the absolute Gibbs free energy of the referenced substances, in kcal/mol, per the electronic and thermal correction terms as calculated by Gaussian 16. The x stands for the number of H atom reductions to the cluster, and all components are implicitly solvated. Experimentally the individual redox events coalesce into 2H^+^/2*e* ^*–*^ PCET events,^75^ as shown in Figure 2, and thus their BDFE­(O–H) is calculated on an average basis for these events per eq 3.

**Figure 2:**
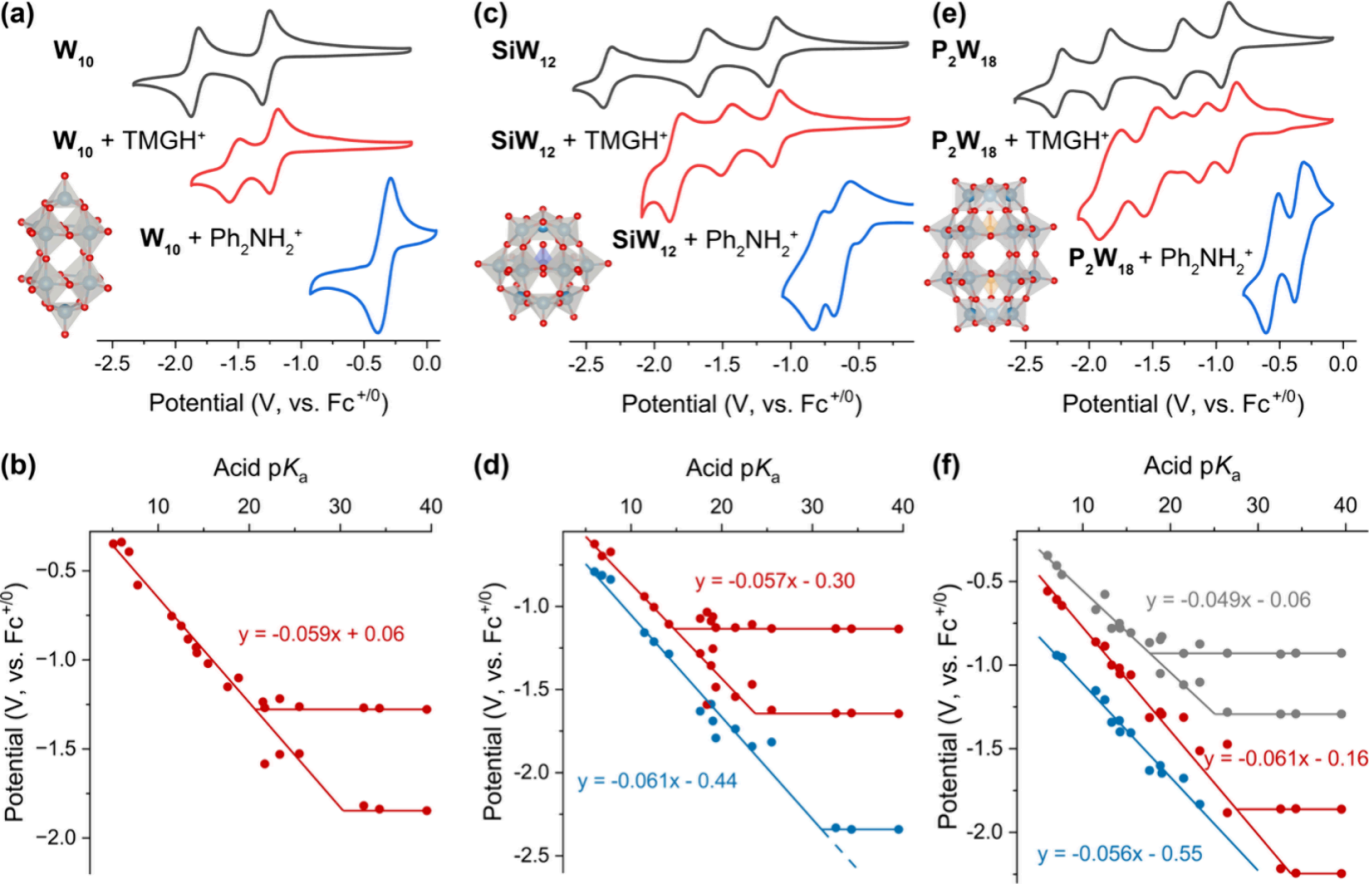
CVs of 1 mM polyoxotungstate cluster (a) **W** **_10_** **O** **_32_** **^–4^**, (c) **SiW** **_12_** **O** **_40_** **^–4^**, and (e) **P** **_2_** **W** **_18_** **O** **_62_** **^–6^** in MeCN with 100 mM [^*n*^Bu_4_N]­PF_6_ at a scan rate of 100 mV/s. Black traces are in the absence of acid, red and blue traces are in the presence of 4 mM 1,1,3,3-tetramethylguanidinium tetrafluoroborate (TMGH^+^, p*K*_a_ = 23.35) and diphenylammonium tetrafluoroborate (Ph_2_NH_2_^+^, p*K*_a_ = 5.98), respectively. Potential-p*K*_a_ diagrams for (b) **W** **_10_** **O** **_32_** **^–4^**, (d) **SiW** **_12_** **O** **_40_** **^–4^**, and (f) **P** **_2_** **W** **_18_** **O** **_62_** **^–6^**. All the potentials are calibrated by using Fc^+/0^ as an internal standard after each measurement. Each data point represents a CV collected in the presence of one organic acid; individual CVs are presented in Figures S2–S4 with corresponding p*K*_a_ values given in Table S2. CVs and a potential-p*K*_a_ diagram for **W** **_6_** **O** **_19_** **^–2^** have been reported previously by Matson; see ref 37 for details.

## Results and Discussion

The thermochemistry of proton-coupled electron transfer (i.e., surface hydroxide BDFE­(O–H) values) for a series of POTs, **W** **_6_** **O** **_19_** **^–2^**, **W** **_10_** **O** **_32_** **^–4^**, **SiW** **_12_** **O** **_40_** **^–4^**, and **P** **_2_** **W** **_18_** **O** **_62_** **^–6^**, were investigated both experimentally and theoretically to elucidate effects from cluster size, shape, and specific H-binding site geometries. Experimental BDFE­(O–H) determinations via potential-p*K*_a_ analysis are presented in Figure 2 (note that potential-p*K*_a_ data for **W** **_6_** **O** **_19_** **^–2^** has previously been reported elsewhere^37^), revealing substantial changes in redox characteristics with each different cluster morphology. The determined reduction potentials, p*K*_a_ analyses, and BDFE­(O–H) values are summarized in Table S1, and BDFE­(O–H)_avg_ values are compared with DFT results in Table 2 (*vide infra*).

Considering the previously reported PCET activity of the **W_6_O_19_^–2^** cluster, no shift was observed for the first (more positive) redox event upon the addition of organic acid, while for the second (more negative) redox event, the peak was convoluted with hydrogen evolution brought about by the acidity.^37^ This indicates that the surface of the Lindqvist-type polyoxotungstate is too acidic to bind hydrogen from the organic acids investigated, and suggests a very low BDFE­(O–H), consistent with our present DFT findings (Table 2).

As illustrated in Figure 2a, electron transfer of **W** **_10_** **O** **_32_** **^–4^** is coupled to proton transfer and the redox events shift anodically along with the proton activity (decreasing p*K*_a_) of the introduced organic acid. In the absence of a proton source, **W** **_10_** **O** **_32_** **^–4^** exhibits two fully reversible reduction events (*E*_1/2_ = −1.28 and −1.85 V, vs Fc^+/0^, black trace in Figure 2a). The CV of **W** **_10_** **O** **_32_** **^–4^** remains unchanged following addition of 4 equiv of organic acids with p*K*_a_ $\underset{\approx }{>}$ 30. Upon addition of 1,1,3,3-tetramethylguanidinium tetrafluoroborate (TMGH^+^, p*K*_a_ = 23.35 in MeCN) into the solution, the most negative redox event shifts anodically to −1.53 V, while the reduction process located at −1.28 V remains at essentially the same value (red trace in Figure 2a). In the presence of stronger organic acids, the two, 1 *e* ^*–*^ reduction events collapse into a 2H^+^/2*e* ^*–*^ process with preserved reversibility. The multielectron/multiproton reduction process continues to shift toward more positive potentials as organic acids with lower p*K*_a_ values are added to solution. For example, the addition of diphenyl ammonium tetrafluoroborate (Ph_2_NH_2_^+^, p*K*_a_ = 5.98 in MeCN) leads to a two-electron redox peak shifting to −0.34 V (blue trace, Figure 2a). A plot of redox potentials of **W** **_10_** **O** **_32_** **^–4^** cluster in the presence of organic acids with p*K*_a_ values ranging from 5 to 40 are compiled into the potential-p*K*_a_ diagram for the assembly given in Figure 2b, illustrating a linear relationship with the slope of ∼ 59 mV/p*K*_a_ unit. The slope is almost identical to the Nernstian 59.2 mV/p*K*_a_ unit, suggesting a two-electron two-proton (ratio 1:1) transfer. The p*K*_a_ values of the **W** **_10_** **O** **_32_** **^–4^** cluster are then estimated by locating the intersections of the acid independent (horizontal lines) and dependent events (diagonal line), affording values of 20.7 ± 2.0 (W_10_O_31_(OH)^−4^ → W_10_O_32_^–5^ + H^+^) and 30.3 ± 3.7 (W_10_O_30_(OH)_2_^–4^ → W_10_O_31_(OH)^−5^ + H^+^). Application of eq 1 calculates individual BDFE­(O–H) values of 51.4 ± 2.7 and 51.4 ± 5.1 kcal/mol for the reduced, protonated **W** **_10_** **O** **_32_** **^–4^**. However, given that the CV indicates a multielectron/multiproton transfer process is operative in this system, we consider only the average BDFE­(O–H) value for the transfer of the first two H atoms as the thermochemical descriptor governing PCET at the surface of the reduced and protonated species.

Analogous experiments were performed for **SiW** **_12_** **O** **_40_** **^–4^** and **P** **_2_** **W** **_18_** **O** **_62_** **^–6^**, and their potential-p*K*_a_ diagrams are presented in Figures 2c & 2d and 2e & 2f, respectively. While prior work from the Matson lab has described the potential-p*K*_a_ diagram for the phosphate-centered Keggin type POT, **PW** **_12_** **O** **_40_** **^–3^**,^27^ we note that it has a different ratio of cluster charge vs the number of tungsten atoms, i.e. different charge/size ratio as compared to **W** **_6_** **O** **_19_** **^–2^** and **W** **_10_** **O** **_32_** **^–4^**, and thus would complicate investigation into cluster morphology by introducing charge effects.^76–78^ Accordingly herein we consider the analogous silicate-centered Keggin cluster **SiW** **_12_** **O** **_40_** **^–4^** and the diphosphate-centered Wells-Dawson **P** **_2_** **W** **_18_** **O** **_62_** **^–6^**, which hold the same cluster charge/W ratio as **W** **_6_** **O** **_19_** **^–2^** and **W** **_10_** **O** **_32_** **^–4^**. We briefly consider the effect of changing the Keggin cluster guest ion below.

In the CV of **SiW_12_O_40_^–4^** in acetonitrile, three one-electron redox events (−2.34, −1.65, and −1.14 V, vs Fc^+/0^) are observed (black trace, Figure 2c). Upon addition of 4 equiv of organic acids with p*K*_a_ values greater than 30, the electron transfer behaviors are acid independent. With the addition of TMGH^+^, three one-electron events turn into one 2-electron and two 1-electron reduction processes at −1.84, −1.47, and −1.11 V, respectively (red trace, Figure 2c). The increase in the total number of reduction events observed is attributed deductively that in nonacidic acetonitrile an additional redox peak with more negative potential must exist outside of the experiment window. In the presence of a moderate acid, the hidden redox event shifts to collapse with the reduction process located at −2.34 V, resulting in a multielectron/multiproton reduction of the assembly. This reduction process shifts anodically in the presence of stronger organic acids (blue points, Figure 2d). Addition of an even stronger acid (Ph_2_NH_2_^+^) triggers the collapse of the remaining two 1-electron events, resulting in a CV with two, 2H^+^/2*e* ^*–*^ events for **SiW_12_O_40_^–4^** (blue trace, Figure 2c). The behavior of two 2H^+^/2*e* ^*–*^ couplings is similar to that previously reported for the **PW_12_O_40_^3–^** cluster.^27^ Two linear correlations are fitted to reveal Nernstian relationships with slopes of −61 and −57 mV/p*K*_a_ unit, with p*K*_a_ for the reduced, protonated variants of the cluster found to be 14.8 ± 3.8 (SiW_12_O_39_(OH)^−4^ → SiW_12_O_40_^–5^ + H^+^), 23.0 ± 4.0 (SiW_12_O_38_(OH)_2_^–4^ → SiW_12_O_39_(OH)^−5^ + H^+^), and 30.3 ± 4.0 (SiW_12_O_37_(OH)_3_^–4^ → SiW_12_O_38_(OH)_2_^–5^ + H^+^). Applying Bordwell eq 1, the BDFE­(O–H)_avg_ values are calculated to be 46.4 ± 5.3 (SiW_12_O_38_(OH)_2_^–4^ → SiW_12_O_40_^–4^ + 2H^•^) and 40.2 ± 5.5 kcal/mol (SiW_12_O_36_(OH)_4_^–4^ → SiW_12_O_38_(OH)_2_^–4^ + 2H^•^). For individual BDFE­(O–H) values for 1H^+^/1e^–^ reduced clusters, see Table S1 in the Supporting Information file. Comparison of these values to the analogous P-centered Keggin cluster **PW_12_O_40_^–3^**, indicates that the change in central [XO_4_] from X = P to X = Si in the case of **SiW_12_O_40_^–4^** does not result in substantial change in the affinity of the surface for H atoms; i.e., similar BDFE­(O–H) values are observed for both clusters: BDFE­(O–H)_avg_ ∼ 48 kcal/mol for **PW_12_O_40_^–3^**, (PW_12_O_38_(OH)_2_^–3^ → PW_12_O_40_^–3^ + 2H^•^) and BDFE­(O–H)_avg_ ∼ 46 kcal/mol for **SiW_12_O_40_^–4^**, (SiW_12_O_38_(OH)_2_^–4^ → SiW_12_O_40_^–4^ + 2H^•^).

The acid-dependent electrochemistry of Wells-Dawson-type **P_2_W_18_O_62_^–6^** is presented in Figures 2e & 2f. **P_2_W_18_O_62_^–6^** exhibits four reversible, one-electron redox events in aprotic electrolyte at −2.25, −1.86, −1.29, and −0.93 V, respectively. With the introduction of 4 equiv of organic acids, the redox events again shift in the anodic direction and collapse in pairs of 1*e* ^–^ events. With the strongest acids, three multielectron-multiproton reduction events are observed. Similar as observed in the case of **SiW_12_O_40_^–4^**, the presence of protons unveils additional reduction events by shifting them into the electrochemically accessible window. Mapping out the potential-p*K*_a_ diagram of **P_2_W_18_O_62_^–6^** indicates four p*K*_a_ values of 17.7 ± 2.8, 25.1 ± 3.8, 27.6 ± 2.5, and 33.8 ± 2.8 (Figure 2f). Adopting Bordwell eq 1 reveals the surface BDFE­(O–H)_avg_ values for the multielectron/multiproton events to be 56.3 ± 4.5 (P_2_W_18_O_60_(OH)_2_^–6^ → P_2_W_18_O_62_^-6^ + 2H^•^) and 47.3 ± 3.6 (P_2_W_18_O_58_(OH)_4_^–6^ → P_2_W_18_O_60_(OH)_2_^–6^ + 2H^•^); individual BDFE­(O–H) values can be found in Table S1 in the Supporting Information file.

The POT clusters under consideration were further investigated in detail with DFT calculations and their optimized structures are shown in Figure 3. The surface tungsten–oxygen bonds in the POTs may be described by both their bond characteristics and their geometric distinction, comprising O_B_ defined by two singly bonded adjacent tungsten atoms, and O_T_ defined by one doubly bonded adjacent tungsten (Figure 3). In total there are five distinct O_B_ site types and two distinct O_T_ site types, with consistent site-type characters across the different clusters.

**Figure 3:**
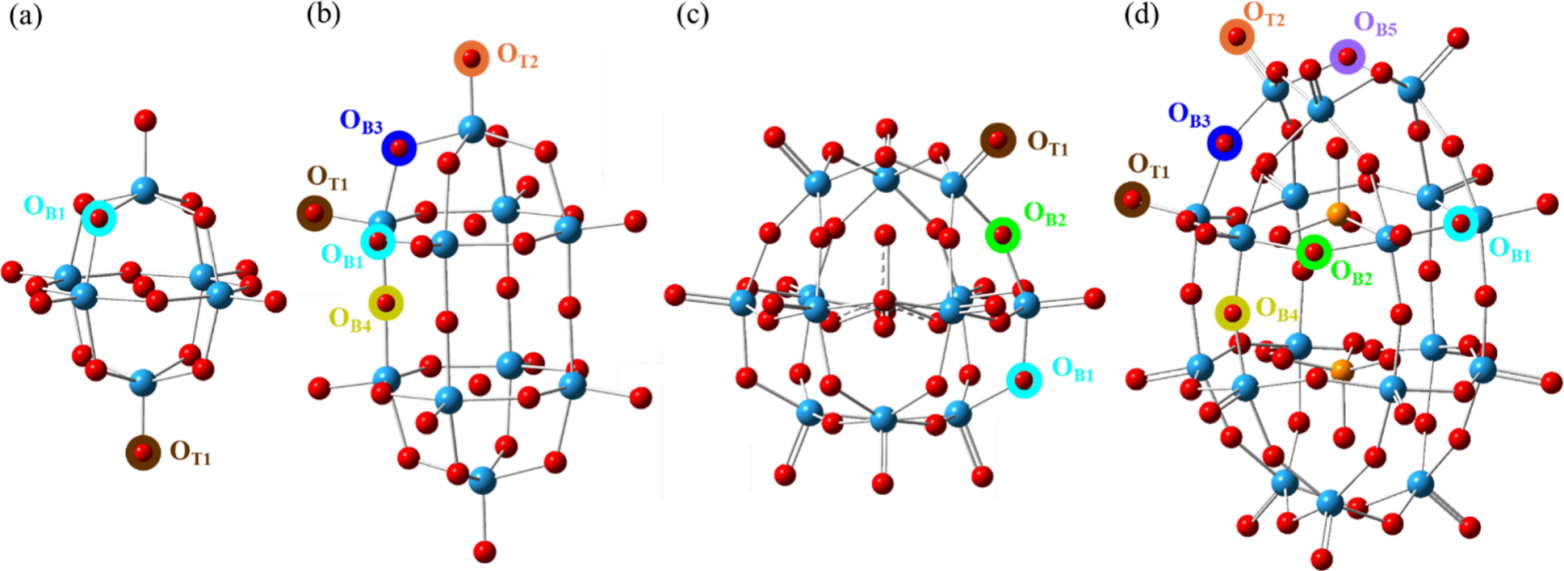
DFT geometrically optimized structures and illustration of symmetrically unique O site locations investigated via DFT for each cluster: (a) **W** **_6_** **O** **_19_** ^–2^, (b) **W** **_10_** **O** **_32_** **^–4^** ,(c) **SiW** **_12_** **O** **_40_** ^–4^, and (d) **P** **_2_** **W** **_18_** **O** **_62_** **^–6^**. Key structural properties of the labeled O_B_ and O_T_ sites are detailed in Table 1. Counterions were removed from Figure 3 for clarity; their positions are illustrated in Figure S1. Atoms key: light blue = W, gray = Si [eclipsed in center of (c)], orange = P, red = O. The guest oxygen (O_G_) are not labeled; they are not surface-accessible for PCET.^24^

A key contrast between the cluster morphologies is apparent where **W** **_10_** **O** **_32_** **^–4^** and **P** **_2_** **W** **_18_** **O** **_62_** **^–6^** both possess one elongated cluster axis, appearing ellipsoidal in shape. By comparison, **W** **_6_** **O** **_19_** **^–2^** and **SiW** **_12_** **O** **_40_** **^–4^** take on tessellated spherical shapes. We note the common conventions to characterize ellipsoidal cluster “cap” and “belt” features^13^ (Figure S5), and to describe the O_B_ sites as being located on asymmetric octahedral “edges” or “corners”, and we denote the features corresponding to each unique O site type in Table S3. **W** **_10_** **O** **_32_** **^–4^** and **P** **_2_** **W** **_18_** **O** **_62_** **^–6^** exhibit distinct structures and O_B_ sites along the lengths of their respective differentiating axes, and their circular belt features are defined by perpendicular intersection with their elongated axes. Conversely, **W** **_6_** **O** **_19_** **^–2^** and **SiW** **_12_** **O** **_40_** **^–4^** do not possess anisotropic axes to distinguish the O_B_ sites, and thus every O_B_ site could be part of belt and/or cap features (Figure S5). DFT computed BDFE­(O–H)_avg_ corresponding to the first 2 hydrogen atom reductions of two of each unique site type as identified in Figure 3 are presented in Table 1.

**Table 1:** DFT BDFE­(O–H)_avg_ Values for Different O Site Types, and Relevant W–O–W Bond Angles

cluster	site type	location description^a^	average W–O–W angle, degrees^b^	number of symmetric sites	DFT 0–2H BDFE(O–H)_avg_, kcal/mol
**W**_**6**_ **O**_**19**_^**–2**^	**O**_**B1**_	belt/cap	120.0°	12	35.2
	**O**_**T1**_	belt/cap		6	24.9
**W**_**10**_ **O**_**32**_^**–4**^	**O**_**T2**_	cap		2	39.3
	**O**_**B3**_	tie	119.8°	8	50.9
	**O**_**B1**_	belt	120.0°	8	38.7
	**O**_**T2**_	belt		8	37.9
	**O**_**B4**_	seam	178.5°	4	28.3
**SiW**_**12**_ **O**_**40**_^**–4**^	**O**_**T1**_	belt/cap		12	35.7
	**O**_**B1**_	belt/cap	124.1°	12	44.7
	**O**_**B2**_	belt/cap	153.0°	12	44.1
**P**_**2**_ **W**_**18**_ **O**_**62**_^**–6**^	**O**_**T2**_	cap		6	40.4
	**O**_**B5**_	cap	125.0°	6	48.6
	**O**_**B3**_	tie	153.2°	12	50.9
	**O**_**B1**_	belt	125.6°	6	44.8
	**O**_**B2**_	belt	154.2°	6	47.9
	**O**_**T1**_	belt		12	40.7
	**O**_**B4**_	seam	164.3°	6	41.5

a Site type labels from Figure 3 are retained. Expanding on the “belt” and “cap” theme commonly employed to describe POM cluster features, we further designate unique O sites of (i) “tie” (as in neck-tie) and (ii) “seam” to precisely identify the (i) O_B3_ between the belt and cap tungsten and (ii) O_B4_ where the different sides of the ellipsoidal POTs are connected.

b Corresponding W–O bonds are detailed in Table S3, including average bond distances, their standard deviations, and average W–O–W bond angles. In general, relative to W–O_B_ distances, W–O_T_ are ∼ 0.2 Å shorter, and W–O_G_ are ∼ 0.4 Å longer. Belt site types are differentiated by their W–O–W bond angles.

Our use of symmetry-unconstrained, counterion-balanced calculations is notable toward precisely characterizing the structures of the studied clusters to develop structure–function relationships dictating the thermochemistry of H atom uptake across the series. To the unaided eye, these clusters evince high degrees of symmetry (Figure 1 and Figure 3) if one considers them without their counterions. Their ideal point groups would be *O_h_* for **W_6_O_19_^–2^**, *D_4h_* for **W_10_O_32_^–4^**, *T*_d_ for **SiW_12_O_40_^–4^**, and *D_3h_* for **P_2_W_18_O_62_^–6^**. However, each optimized cluster structure adopts subtle differences in bond lengths distorting their symmetry (Table S3), and they exhibit spontaneous relaxation energies between 0.3–2 kcal/mol in magnitude vs symmetric structures (Table S4). This finding aligns with previous work by Yan et al. on analogous molybdenum Lindqvist, Keggin, and Wells-Dawson cluster anions, who found that the Mo cluster alternating bond length distortions arose from second order pseudo Jahn–Teller effects,^79^ and conclusions by Bersuker that spontaneous symmetry breaking is invariably indicative of some Jahn–Teller phenomena.^80,81^ Additionally, Poblet et al. have suggested that solvent and counterion effects need to be considered to precisely resolve the preferred active sites for H-binding.^82^

Considering distinct site geometries and their DFT BDFE­(O–H) (Table 1), one notices a pattern that when the O_B_ sites are differentiated in the ellipsoidal clusters, the O_B3_ tie sites are thermodynamically favored. In contrast, the O_B4_ seam sites are significantly disfavored, and the belt sites O_B1_ and O_B2_ are disfavored in comparable magnitude as the O_T_ sites. Examining the ellipsoidal W–O bond lengths of the cluster structures including counterions (Table S3), one notes that there is significantly less variance in the belt W–O_B1_ and W–O_B2_ bonds than in the W–O_B3_ bonds. This suggests that symmetry breaking preferentially involves the O_B3_ tie sites and their asymmetric W–O bonds, one connected to a belt tungsten and the other to a cap. Concomitantly, the belt W–O bonds retain a high degree of symmetry. We hypothesize that this patterned way the ellipsoidal clusters break from their ideal symmetry around the O_B3_ sites may preferentially minimize bond distortions in the belt, stabilize the belt feature geometries, and lower their LUMO energies, thus increasing the nucleophilicity of O_B3_ sites. This is consistent with previous work by Zhang et al. on the stability of different Wells-Dawson POT isomers, who found that although differences in W–O bonds were small, the degree of associated distortions correlated directly to the relative instabilities of the clusters.^83^ By contrast, the spherical clusters do not show significant differences in their W–O bond length variances (Table S3), nor in the difference between O_B1_ and O_B2_ BDFE­(O–H) (Table 1). **SiW** **_12_** **O** **_40_** **^–4^** shows marginally higher standard deviation of the O_B2_ W–O–W bond angle than others (Table S3), indicating that some symmetry breaking has been favored and may provide some smaller degree of stabilization to the O_B1_ sites (Table 1) despite the cluster’s interconnected belt constraints.

The highest DFT BDFE­(O–H)_avg_ site locations for each cluster (sites of thermodynamically preferred hydrogen binding), are paired and compared with experimental BDFE­(O–H)_avg_ values in Table 2. Additionally, Table 2 includes the results of DFT calculations performed for at least one additional BDFE­(O–H) beyond the experimental data, on the same distinct site types found to align with the first experimental BDFE­(O–H)_avg_. Incremental 2H reductions at other O site types were found to be less thermodynamically favored. Individual nonaveraged BDFE­(O–H) values are provided in Tables S1 and S5 for experiment and DFT, respectively. We find that the experimental results correspond to the theoretical calculations for the favored O_B_ sites as opposed to O_T_ locations (Tables 1 and 2), suggesting that H atom uptake occurs at bridging oxide sites at the surface of the assembly. In the case of the ellipsoidal shaped structures, **W** **_10_** **O** **_32_** **^–4^** and **P** **_2_** **W** **_18_** **O** **_62_** **^–6^**, the alignments of the combined results suggest a preference for H atom uptake at bridging oxide sites located between belt and cap features (O_B3_, see Figure 3). Excellent parity is achieved between the experimental and corresponding site-specific DFT BDFE­(O–H)_avg_ values (Figure 4a), with 91% trend-capture. For the absolute BDFE­(O–H)_avg_ values, the attained root mean squared error (RMSE) of 2.8 kcal/mol vs parity is reasonable for this complex electrochemical system. All but one DFT BDFE­(O–H)_avg_ are well within the experimental uncertainty windows, with the exception comprising the first two BDFE­(O–H) values of **P** **_2_** **W** **_18_** **O** **_62_** **(OH)** **^–6^** which are still within 1 kcal/mol of their experimental uncertainty windows (Tables 2, S1, S5). Via DFT calculation, O_T_ sites were found to afford BDFE­(O–H)_avg_ about 10 kcal/mol lower than the preferred O_B_ sites.

**Table 2:** BDFE­(O–H)_avg_ Values Determined Via Experiment and Theory^a^

Cluster	BDFE(O–H)_avg_ for #H:	Experimental BDFE(O–H)_avg_, kcal/mol	DFT BDFE, kcal/mol	Site Type
**W**_**6**_ **O**_**19**_^**–2**^	1 and 2	not observed	35.2	**O**_**B1**_
**W**_**10**_ **O**_**32**_^**–4**^	1 and 2	51.4	50.9	**O**_**B3**_
	3 and 4	not observed	35.6	
**SiW**_**12**_ **O**_**40**_^**–4**^	1 and 2	46.4	44.7	**O**_**B1**_
	3	40.2	39.2	
	4	not in range	37.6	
**P**_**2**_ **W**_**18**_ **O**_**62**_^**–6**^	1 and 2	56.3	50.9	**O**_**B3**_
	3 and 4	47.3	45.9	
	5 and 6	not in range	41.2	

a Site type labels from Figure 3 are retained. DFT values are in great agreement with experimental; parity is further assessed with Figure 4a. 1H incremental (non-averaged) BDFE­(O–H) values are provided in Table S5.

The observation that O_B_ sites are preferred for H atom uptake is in alignment with work by our groups on ligated Lindqvist polyoxovanadate clusters, where the O_B_ sites were found to be thermodynamically favored as compared to O_T_.^22^ Earlier theoretical works on cation interactions with Keggin and Wells-Dawson anions by López et al.^84^ and Fernández et al.^49^ are also consistent with this conclusion. However, the result indicating the preference of proton uptake at O_B3_ on **W** **_10_** **O** **_32_** **^–4^** appears in contrast to that of Ravelli et al., who found that O_T1_ would be the favored 1H binding site by 0.53 kcal/mol.^47^ However, the optimization Ravelli et al. performed was symmetry constrained and without counterions, vs counterion inclusion and unrestricted geometry optimization in the present work. Presently, 1H binding to the same O_T1_ site with the same orientation used by Ravelli et al. is found 9 kcal/mol higher in energy than the favored O_B3_ sites, although similar in energy to the O_B1_ belt sites. “Edge” and “corner” designations distinguish O_B1_ from O_B2_ (Table S3, Figure 3), but we did not note any specific energetic correlations with “edge” and/or “corner” descriptions. Similar to previous results on different systems,^9,22,85^ the BDFE­(O–H)_avg_ values examined are found to decrease with increasing degree of H reduction (Tables 2, S1, S5, and Figure S6).

A depiction of the impact of POT morphology on BDFE­(O–H) is provided in Figure 4b, where both the site-average O_B_ (light-blue dashed line) and O_T_ (yellow dotted line) DFT-calculated BDFE­(O–H) correlate linearly with the formal W oxidation state for the 2H reduced clusters. Here the increasing formal W oxidation state for the same 2H degree of reduction serves as a descriptor of cluster size. For **W** **_10_** **O** **_32_** **^–4^** and **P** **_2_** **W** **_18_** **O** **_62_** **^–6^**, one notes that the distinct O_B_ sites possess the same formal oxidation state, but different BDFE­(O–H). Thus, the BDFE­(O–H) for these O_B_ appear as differentiated vertical points. For the ellipsoidal clusters this differentiation is substantial, while for spherical **SiW** **_12_** **O** **_40_** **^–4^**, the O_B1_ and O_B2_ sites are barely distinguishable in energy. The O_T_ sites exhibit less differentiation than the O_B_ sites. Similar trends exist for the 1H reduced clusters (Figure S7), and although higher degrees of cluster reduction give rise to complications with changing BDFE­(O–H) (Figure S6), the 1H and 2H trends connect directly to the experimentally investigated BDFE­(O–H)_avg_. The Figure 4b size and shape trends were further scrutinized via employ of an alternative basis set. The alternative def2-TZVP/SVP basis set (see Computational Methods) corroborated the BDFE­(O–H) trend vs cluster size and differentiation with anisotropic cluster shape (Figures S8, S9a); however, the LANL2DZ basis afforded superior parity with experiments (Figures 4a, S9). Considering the combination of size and shape effects, the **W** **_10_** **O** **_32_** **^–4^** cluster exhibits a larger magnitude of differentiation than the **P** **_2_** **W** **_18_** **O** **_62_** **^–6^** cluster (Figure 4b). The larger differentiation could align with the larger length/diameter ratio of the **W** **_10_** **O** **_32_** **^–4^** cluster, although the suggested behavior from these data points is somewhat anecdotal.

**Figure 4:**
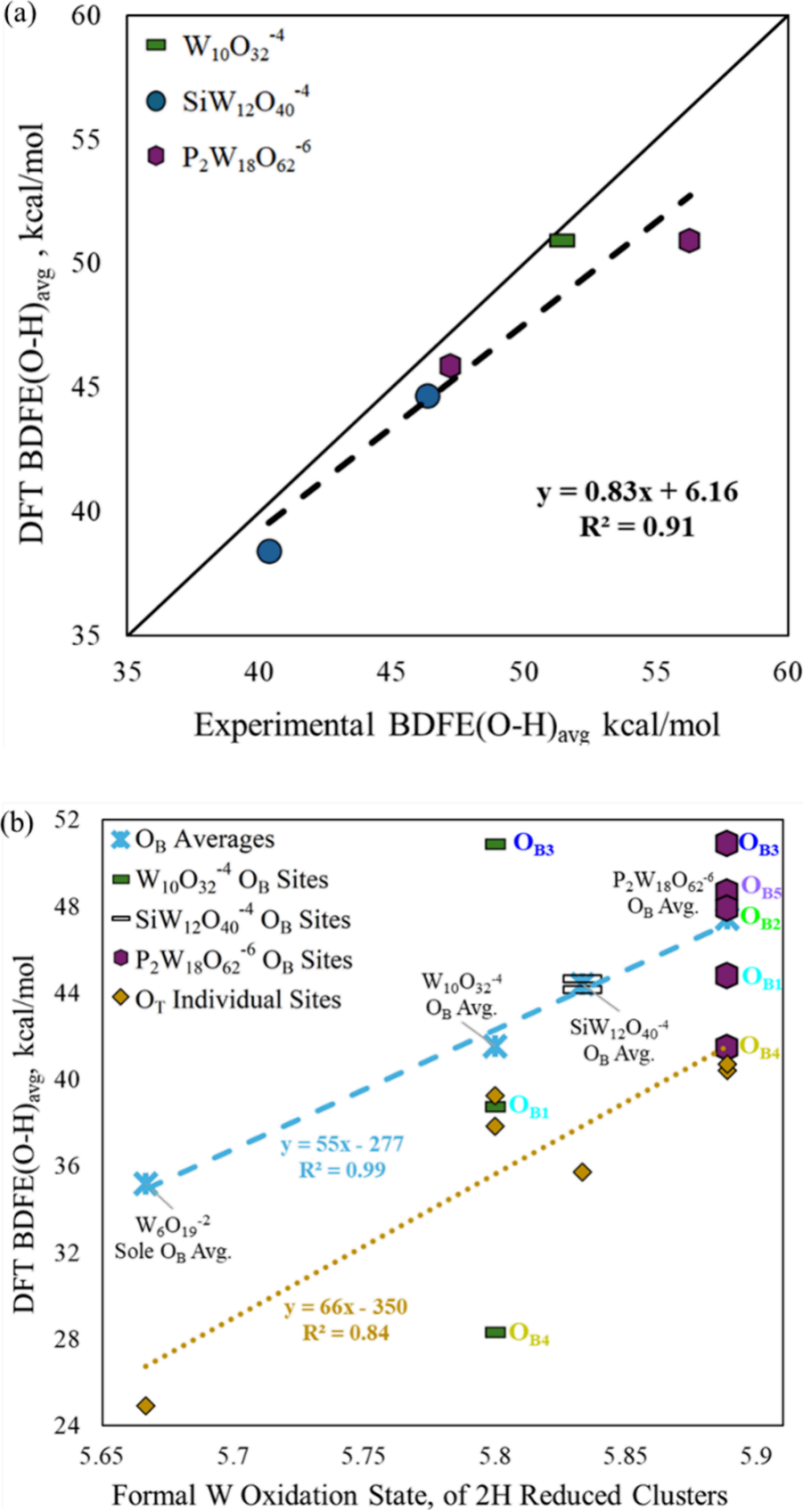
(a) Comparison of DFT-calculated vs experimental BDFE­(O–H)_avg_ values of different POTs. (b) Relationship between DFT-calculated BDFE­(O–H) values and formal W oxidation states of 2H reduced POT clusters for different O_B_ site types (locations as described in Figure 3), their per-cluster site-weighted averages (light blue asterisks), and individual O_T_ sites (gold diamonds). The average formal W oxidation states of the 2H reduced clusters serve as a proxy for cluster size.

Previous work by our groups found NBO^71^ atomic charges instructive in rationalizing BDFE­(O–H) trends.^22^ Accordingly, the present BDFE­(O–H) data were explored for correlations with atomic charges, across different clusters, different sites, and different extents of H reduction. Various correlations were uncovered regarding per-cluster averages (Figure S10a), site-specific atomic charges (Figure S10b), their combination (Figure 5a), and the aforementioned tungsten formal oxidation states (Figure 4b, blue and yellow lines). Similar to our prior work,^22^ correlation was also found between the BDFE­(O–H) and O_B_ charges of H atom reduced clusters (Figure S11). None of these trends were found to be universal across different clusters as well as across all site types. However, this may be due to different natures of the cluster differentiations. For example, the trend of DFT BDFE­(O–H) vs O_B_ charges is obfuscated when all site-specific values are represented (Figure S12). Considering O_B_ vs O_T_ sites, on a site-type-average basis we find that the BDFE­(O–H) is higher with the more negative O_B_ charges (Figure S13). As the O_T_ charges are much less negative, the O_T_ BDFE­(O–H) values do not follow the same trends with the O_B_ BDFE­(O–H) vs atomic charges.

**Figure 5:**
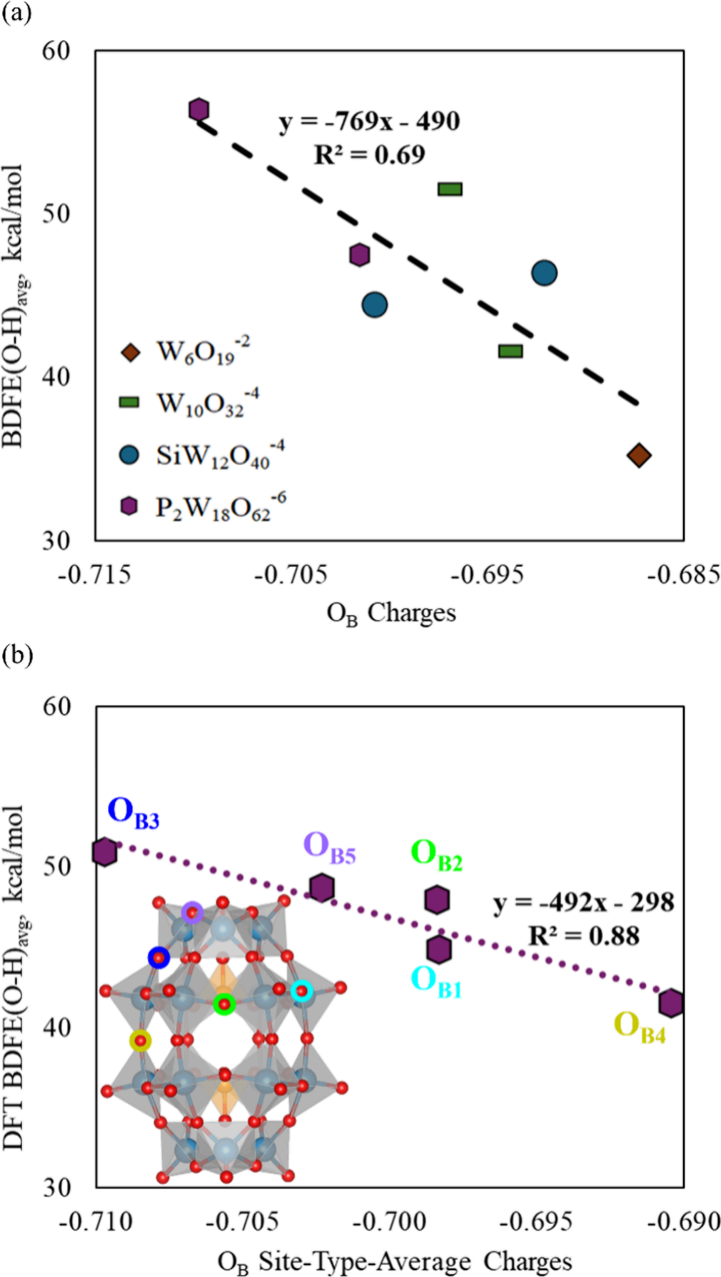
BDFE­(O–H) as a function of O_B_ charges for unreduced clusters: (a) DFT and experimental values vs cluster-average and specific site-type average charges, respectively (separate plots provided in Figure S9), and (b) DFT BDFE­(O–H) values vs site-specific O_B_ charges for unreduced **P** **_2_** **W** **_18_** **O** **_62_** **^–6^**.

Considering the charge-BDFE­(O–H) relationships for the differentiated O_B_ of the ellipsoidal clusters, we find that a similar relation holds for **P** **_2_** **W** **_18_** **O** **_62_** **^–6^** with the unreduced O_B_ charges (Figure 4b) as with the trends per unreduced cluster site-weighted averages (Figure S10a) and particularly compared to the experimental trend with the thermodynamically favored O_B_ sites (Figure S10b). However, this charge relation is not present in the differentiated O_B_ of **W** **_10_** **O** **_32_** **^–4^** (Figure S14).

To better understand the geometric differentiation of the ellipsoidal site BDFE­(O–H) values we plotted the cluster frontier molecular orbitals, first as afforded by the Kohn–Sham self-consistent field solutions and evaluated their relations to the favored O_B_ sites. For each cluster, we found sets of nearly isoenergetic HOMOs and LUMOs, generally indicating 1 LUMO per W for the spherical clusters or 1 LUMO per belt W for the ellipsoidal clusters, and corresponding numbers of HOMOs (Figures S15–18). Most of the ellipsoidal cluster HOMOs and LUMOs suggest localization of the frontier orbitals on O_B3_ sites and belt W of the ellipsoidal clusters (representative orbitals illustrated in Figure S19). However, previous work by Keith et al. has highlighted that single-determinant Kohn–Sham orbitals are nonunique, and density difference calculations (between differently charged but isostructural clusters) are required to fully resolve orbital natures, including HOMO and LUMO orbitals.^72^ Indeed, some of the frontier orbitals in our calculations would have suggested orbital localizations on less-favored O_B_ and W sites, in contrast to current findings as well as orbital localizations found by others.^44,47,51,53,82,84,86,87^ Thus, we have determined the isostructural electronic density differences corresponding more precisely to each cluster’s LUMO (Figure 6) and HOMO (Figure S20). The positive density difference from a 1*e* ^*–*^ reduction of the neutral cluster describes the LUMO (specific charge states detailed in Figure 6 caption), while the positive density difference from 1*e* ^*–*^ reduction of the +1 charged cluster describes the HOMO (specific charge states detailed in Figure S20 caption), and the accompanying negative density differences describe the reoptimization of existing electron density upon these reductions (Figure S21). For all clusters, we found that the thermodynamically favored O_B_ sites correspond to higher density LUMO and HOMO locations (Figure 6 and Figure S20). For the ellipsoidal clusters, we found that the LUMOs specifically center on the belt W and include the thermodynamically favored adjacent O_B3_ (Figure 6). For all clusters, in both the Kohn–Sham and density difference LUMOs, the localizations on the tungsten centers are observed to hybridize between W_d_ and O_p_ orbitals per their constituent characters, and notably for **W** **_10_** **O** **_32_** **^–4^** and **P** **_2_** **W** **_18_** **O** **_62_** **^–6^** this hybridization occurs with the favored O_B3_ sites. These localizations align with previous findings by others.^84,87,88^ Ravelli et al. also found the belt tungsten to be the preferential recipient LUMO sites under electronic excitation.^47^ The LUMOs of the ellipsoidal clusters make up 2 delocalized favored belt orbital features on either side of their symmetry planes (Figures 6d and 6h), consistent with the findings of Bridgeman and Cavigliasso.^51^ By contrast, the LUMOs of the spherical clusters are degenerate with the cluster symmetries, resulting in equivalent O_B1_ and O_B2_ sites, each having equal BDFE­(O–H) values upon protonation. Thus, the HOMOs and LUMOs elucidated by electronic density differences correlate with the existence and lack of significant BDFE­(O–H) differentiation between ellipsoidal and spherical clusters, respectively, as well as the favoring of O_B3_ sites on ellipsoidal clusters. Hence, we find that the resolution of the LUMOs via electronic density differences serves to indicate thermodynamically favored binding sites of geometrically differentiated systems.

**Figure 6:**
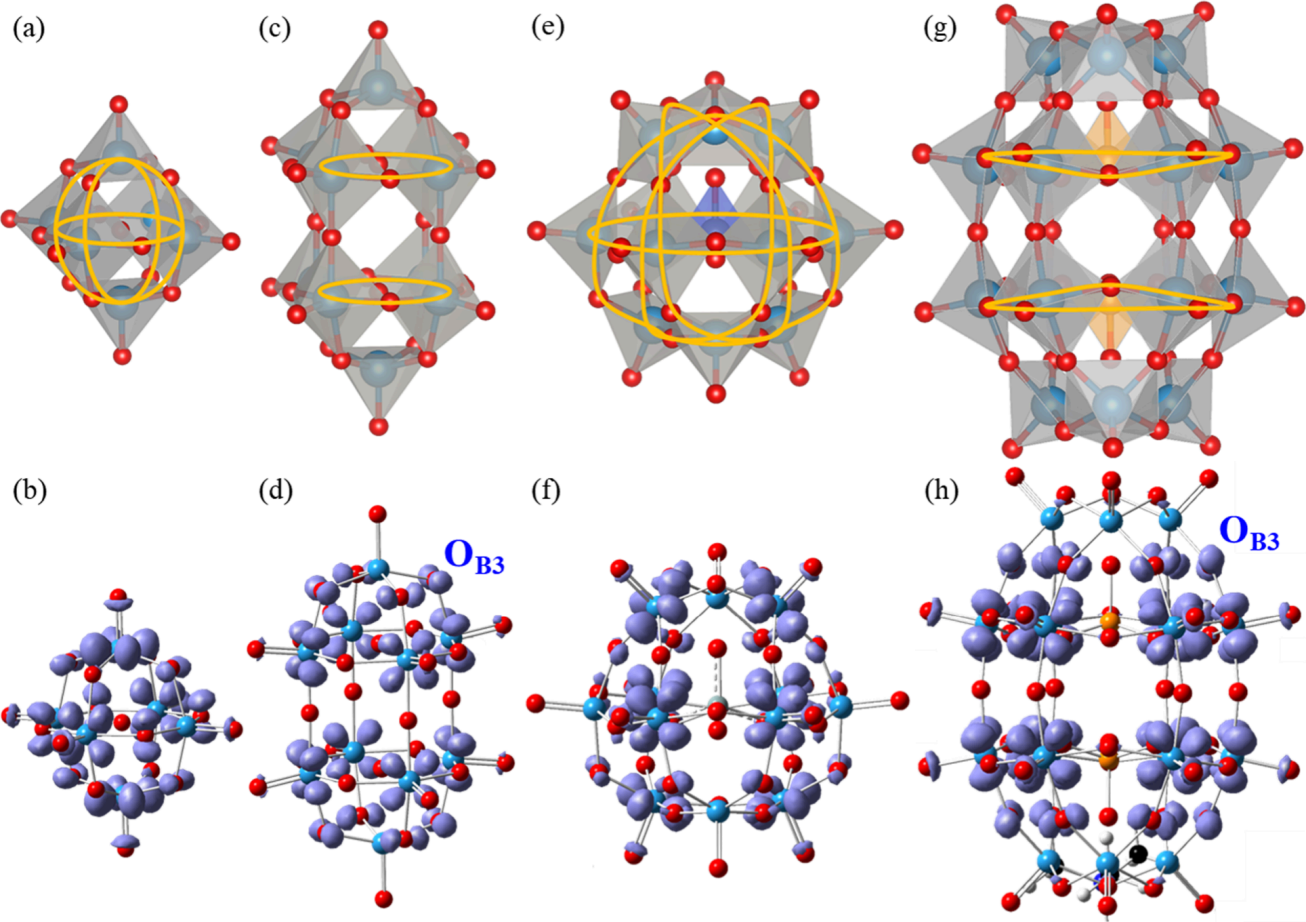
Belt feature identification (yellow) in the cluster structures of (a) **W** **_6_** **O** **_19_** **^–2^**, (c) **W** **_10_** **O** **_32_** **^–4^**, (e) **SiW** **_12_** **O** **_40_** **^–4^**, and (g) **P** **_2_** **W** **_18_** **O** **_62_** **^–6^**; and positive density differences upon isostructural 1*e* ^–^ reduction (purple, *e* ^–^ density gained) for clusters (b), (d), (f), and (h), respectively. Distinct LUMO W belts are apparent in **W** **_10_** **O** **_32_** **^–4^**, and **P** **_2_** **W** **_18_** **O** **_62_** **^–6^**, and hybridize with the favored O_B3_. Counterions were removed from the figures for clarity. Corresponding negative density differences are illustrated in Figure S21 (areas *e* ^–^ density moved away from). Representative contributing molecular orbitals are illustrated in Figure S19.

The Matson group has previously established connection between BDFE­(O–H) thermodynamics and kinetics,^24,32^ and demonstrated that the low-BDFE­(O–H) **PW** **_12_** **O** **_40_** **^–3^** may serve as a selective hydrogenation catalyst.^27^ Similarly, one may expect that higher POM interaction strengths with hydrogen as described by the BDFE­(O–H) can influence chemistries such as C–H activation, where the hydrogen atom binding energy is a descriptor for alkane dehydrogenation kinetics.^89^ Thus, tailoring POT designs to target specific BDFE­(O–H) for a given hydrogen transfer reaction may be realized by optimizing the cluster size to increase or decrease the BDFE­(O–H). Cluster elongation could be employed if even higher BDFE­(O–H) values are desired, or anisotropy may be avoided to minimize the BDFE­(O–H) heterogeneity and thus maximize the number of active sites per W mass.

## Conclusions

In this work we investigated in detail the hydroxide bond dissociation free energies of polyoxotungstates across varying cluster size, shape, and binding sites. We combined electrochemical experiments and DFT calculations to determine the BDFE­(O–H) for the POT clusters **W** **_6_** **O** **_19_** **^–2^**, **W** **_10_** **O** **_32_** **^–4^**, **SiW** **_12_** **O** **_40_** **^–4^**, and **P** **_2_** **W** **_18_** **O** **_62_** **^–6^**, revealing an excellent agreement between theory and experiments on the reported values. We demonstrate that cluster-average BDFE­(O–H) increases linearly with cluster size, with an effective size descriptor being the formal tungsten oxidation state for clusters reduced by two hydrogen atoms. DFT calculations revealed a differentiation of the BDFE­(O–H) values per distinct oxygen binding site types according to cluster morphology, and determined the BDFE­(O–H) of **W** **_6_** **O** **_19_** **^–2^** which was experimentally inaccessible. The DFT results agree with experimental findings of increased acidity for the smaller and more symmetric clusters. For all the clusters examined herein, bridging oxygen sites are thermodynamically favored for H-binding over terminal oxygen. Isotropic clusters such as **W** **_6_** **O** **_19_** **^–2^** and **SiW** **_12_** **O** **_40_** **^–4^** showed essentially equivalent BDFE­(O–H) within their sets of possible O_B_ binding sites. In the ellipsoidal shaped clusters **W** **_10_** **O** **_32_** **^–4^** and **P** **_2_** **W** **_18_** **O** **_62_** **^–6^**, the introduction of an elongated cluster axis allows for O site differentiation, through variation of W–O bond lengths, and emergence of belt features perpendicular to the elongation, which help identify favored and disfavored O_B_ sites for H-binding. Site-specific BDFE­(O–H) thermodynamics were shown to correlate to atomic charges and frontier orbital localizations. The revealed relationships indicate that larger clusters effect higher BDFE­(O–H) and that more symmetric clusters afford more homogeneity in site BDFE­(O–H) values.

Overall, we demonstrate that POT cluster morphology is linked to its electronic properties, which are size and shape dependent, and thereby to the thermodynamics of hydrogen interaction with the clusters. The latter was captured through the BDFE­(O–H) property, which is a known descriptor for reactivity in PCET involving chemistries. Developing such structure–function relationships can help identify novel polyoxometalate structures of interest for selective hydrogen atom transfer chemistries.

## Supplementary Material





## Notes

Experimental data including cyclic voltammetry data corresponding to data points in potential-p*K*_a_ diagram; computational results of DFT calculated structures, bond distances, BDFE­(O–H), molecular orbitals, spontaneous symmetry breaking energies and atomic charges (PDF)
DFT geometrically optimized structures and spatial mapping in formatted checkpoint files, consolidated (ZIP)
