# Supplementary material for: Morphology Effects on Free Energies of Proton-Coupled Electron Transfer in Polyoxotungstates
Source: Inorg Chem. 2025 Oct 23;64(49):23834–45. doi: 10.1021/acs.inorgchem.5c02910 (PMC12709570; doi:10.1021/acs.inorgchem.5c02910)
Supplement: Supplementary file 1 [file ic5c02910_si_001.pdf]

Supporting Information

Morphology effects on free energies of proton-coupled electron transfer in polyoxotungstates

Andreas Towarnicky[1], Zhou Lu[2], Ellen M. Matson[2,*], and Giannis Mpourmpakis[1,3,*]

[1]Department of Chemical Engineering, University of Pittsburgh, Pittsburgh, PA 15261 USA

*Corresponding author e-mail: gmpourmp@pitt.edu

[2]Department of Chemistry, University of Rochester, Rochester, NY 14627 USA

*Corresponding author e-mail: matson@chem.rochester.edu

[3]School of Chemical Engineering, National Technical University of Athens (NTUA), Athens, GR-15780, Greece

Supporting Information Table of Contents

Figure S1. Counterion locations with DFT geometrically optimized structures S3

Table S1. Experimentally determined pK_a values, redox potentials, and BDFE(O–H) S3

Figure S2. Cyclic voltammograms of 1 mM $\mathbf{W_{10}O_{32}^{-4}}$ S4

Figure S3. Cyclic voltammograms of 1 mM $\mathbf{SiW_{12}O_{40}^{-4}}$ S5

Figure S4. Cyclic voltammograms of 1 mM $\mathbf{P_2W_{18}O_{62}^{-6}}$ S6

Table S2. pK_a values of various organic acids in acetonitrile S7

Figure S5. Polyhedra representations of $\mathbf{SiW_{12}O_{40}^{-4}}$ S8

Table S3. Optimized W–O bond lengths per DFT calculations S8

Table S4. DFT symmetry-breaking energies for each cluster without counterions S9

Table S5. DFT-calculated BDFE(O–H) per 1H incremental reductions S9

Figure S6. DFT-calculated BDFE(O–H) vs. degree of H reduction S10

Figure S7. DFT BDFE(O–H) vs. formal W oxidation states of 1H reduced clusters S11

Figure S8. DFT BDFE(O–H) calculated with alternative basis set vs. formal W oxidation states of 2H reduced clusters S11

Figure S9. Parity of differing DFT basis set BDFE(O–H) results, and of alternative DFT basis set calculations vs. experiments S12

Figure S10. Individual relations of DFT and Experimental BDFE(O–H) vs. O_B charges (unreduced clusters) S12

Figure S11. DFT BDFE(O–H) vs. O_B charge for H-reduced clusters S13

Figure S12. Site-specific DFT BDFE(O–H) vs. O_B charges (unreduced clusters) S13

Figure S13. O_B and O_T site-specific DFT BDFE(O–H) vs. O charges (unreduced clusters) S14

Figure S14. $\mathbf{W_{10}O_{32}^{-4}}$ site-specific DFT BDFE(O-H)$_{avg}$ vs. O_B charges (unreduced clusters) S14

Figure S15. Molecular orbital energies for $\mathbf{W_6O_{19}^{-2}}$ S15

Figure S16. Molecular orbital energies for $\mathbf{W_{10}O_{32}^{-4}}$ S15

Figure S17. Molecular orbital energies for $\mathbf{SiW_{12}O_{40}^{-4}}$ S16

Figure S18. Molecular orbital energies for $\mathbf{P_2W_{18}O_{62}^{-6}}$ S16

Figure S19. Representative molecular orbitals for each cluster S17

Figure S20. HOMO positive density difference plots (e^- density gained) S18

Figure S21. HOMO and LUMO negative density difference plots (e^- density lost) S19

References S20

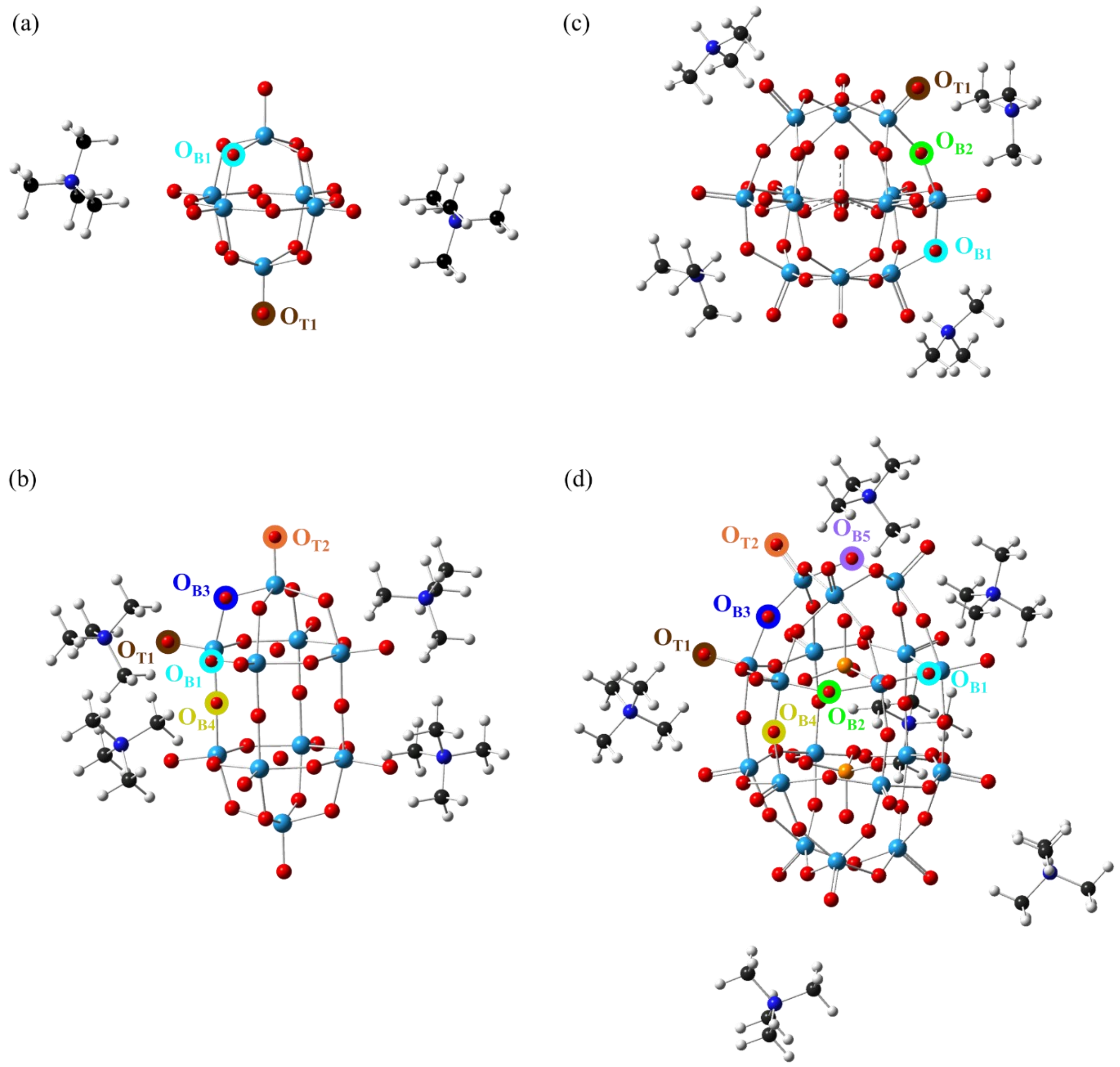

Figure S1. Counterion locations with the same cluster structures and orientations of main text Figure 3, for (a) $\mathbf{W_6O_{19}^{-2}}$, (b) $\mathbf{W_{10}O_{32}^{-4}}$, (c) $\mathbf{SiW_{12}O_{40}^{-4}}$, and (d) $\mathbf{P_2W_{18}O_{62}^{-6}}$. Counterion locations are evenly balanced between the foreground and background.

Table S1. Experimentally determined pK_a values, redox potential ($E_{1/2}$ in V, vs. $Fc^{+/0}$), and corresponding bond dissociation free energy (BDFE in kcal mol^{-1}) of POTs studied in this work.

	pK_a	$E_{1/2}$ (V, vs. $Fc^{+/0}$)	BDFE (kcal mol^{-1})
$\mathbf{W_6O_{19}^{-2}}$ **[per 1]**	n.a.	-1.36	n.a.
	n.a.	-2.32	n.a.
$\mathbf{W_{10}O_{32}^{-4}}$	20.7±2.0	-1.28	51.4±2.7
	30.3±3.7	-1.85	51.4±5.1
$\mathbf{SiW_{12}O_{40}^{-4}}$	14.8±3.8	-1.14	46.6±5.3
	23.0±4.0	-1.65	46.1±5.8
	30.3±4.0	-2.34	40.2±5.5
$\mathbf{P_2W_{18}O_{62}^{-6}}$	17.7±2.8	-0.93	55.4±3.8
	25.1±3.8	-1.29	57.2±5.2
	27.6±2.5	-1.86	47.5±3.4
	33.8±2.8	-2.25	47.0±3.8

n.a.: not applicable.

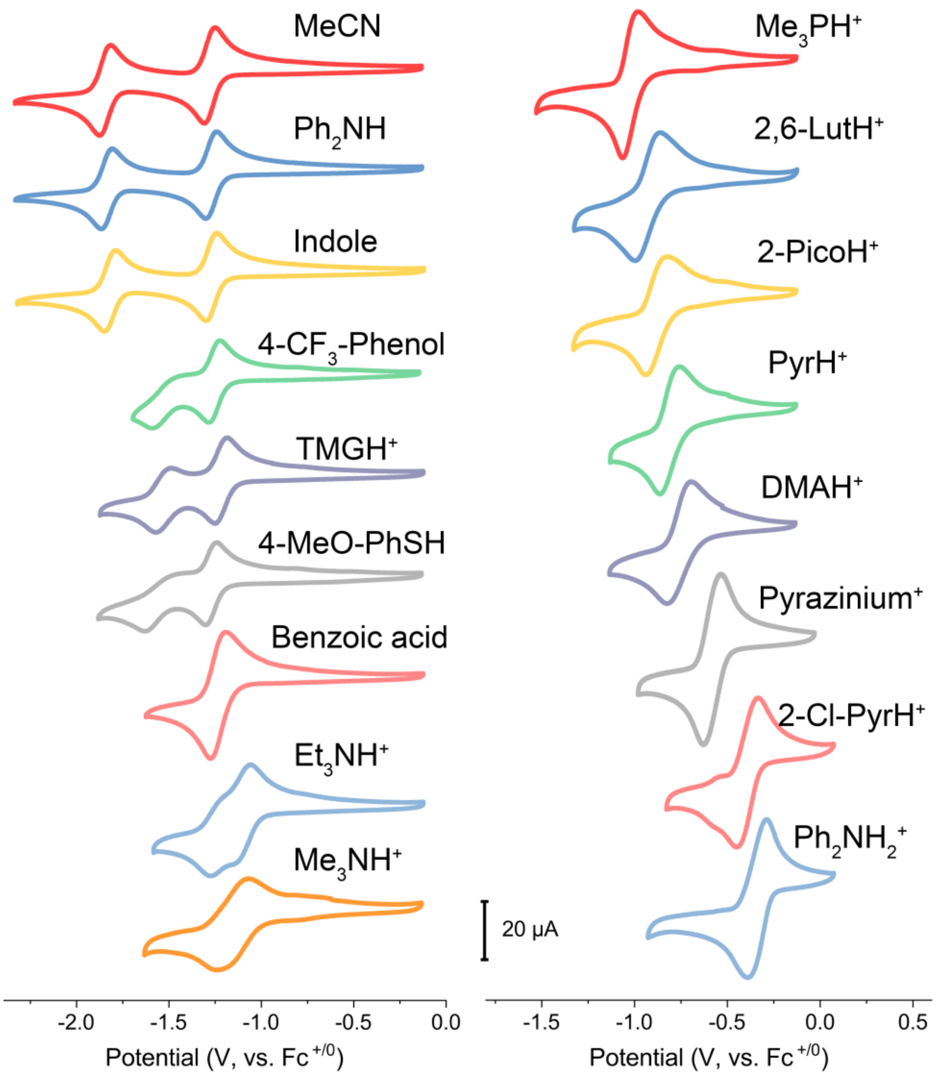

Figure S2. Cyclic voltammograms of 1 mM $\mathbf{W_{10}O_{32}^{-4}}$ obtained in acetonitrile in the presence of 2 mM various organic acids with the scan rate of 100 mV/s, using 0.1 M [^{n}Bu$_4$N]PF$_6$ as the supporting electrolyte. Ferrocene is used for each measurement as the internal standard. The corresponding acids are listed in Table S2.

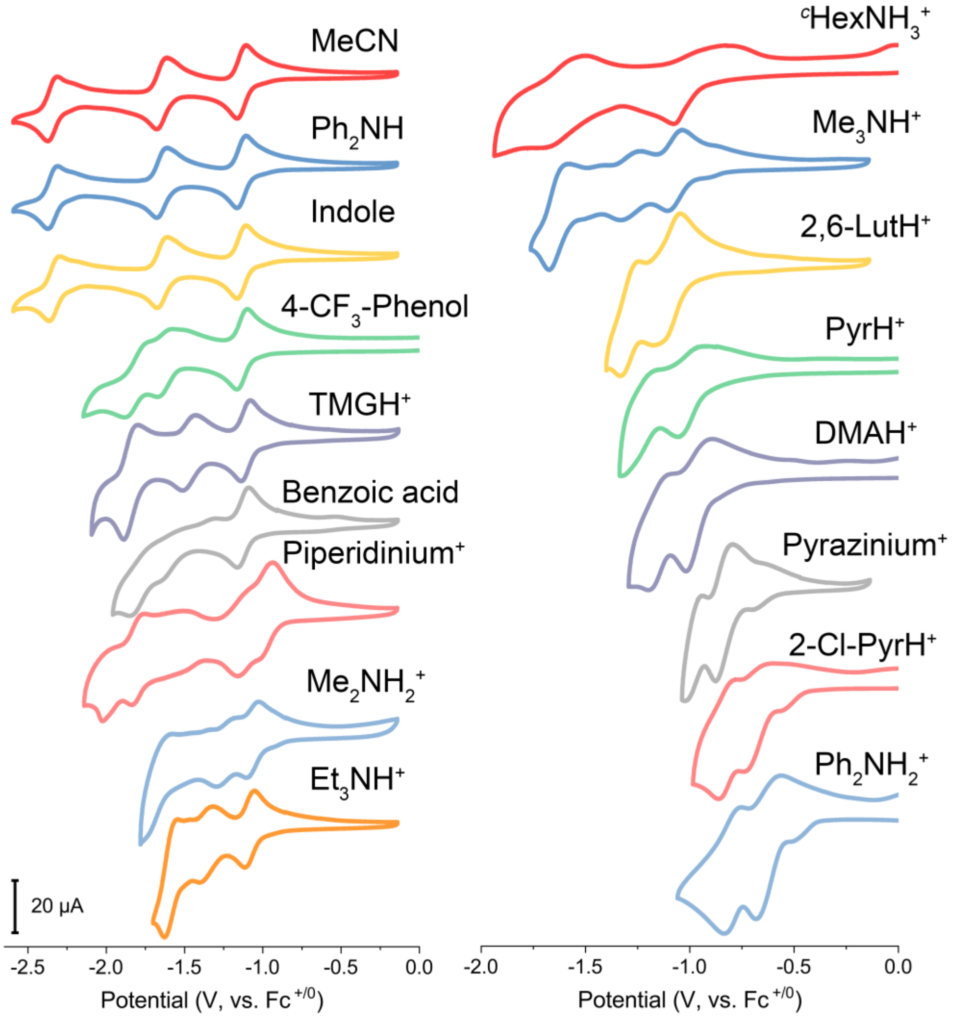

Figure S3. Cyclic voltammograms of 1 mM $\mathbf{SiW_{12}O_{40}^{-4}}$ obtained in acetonitrile in the presence of 4 mM various organic acids with the scan rate of 100 mV/s, using 0.1 M [^{n}Bu$_4$N]PF$_6$ as the supporting electrolyte. Ferrocene is used for each measurement as the internal standard. The corresponding acids are listed in Table S2.

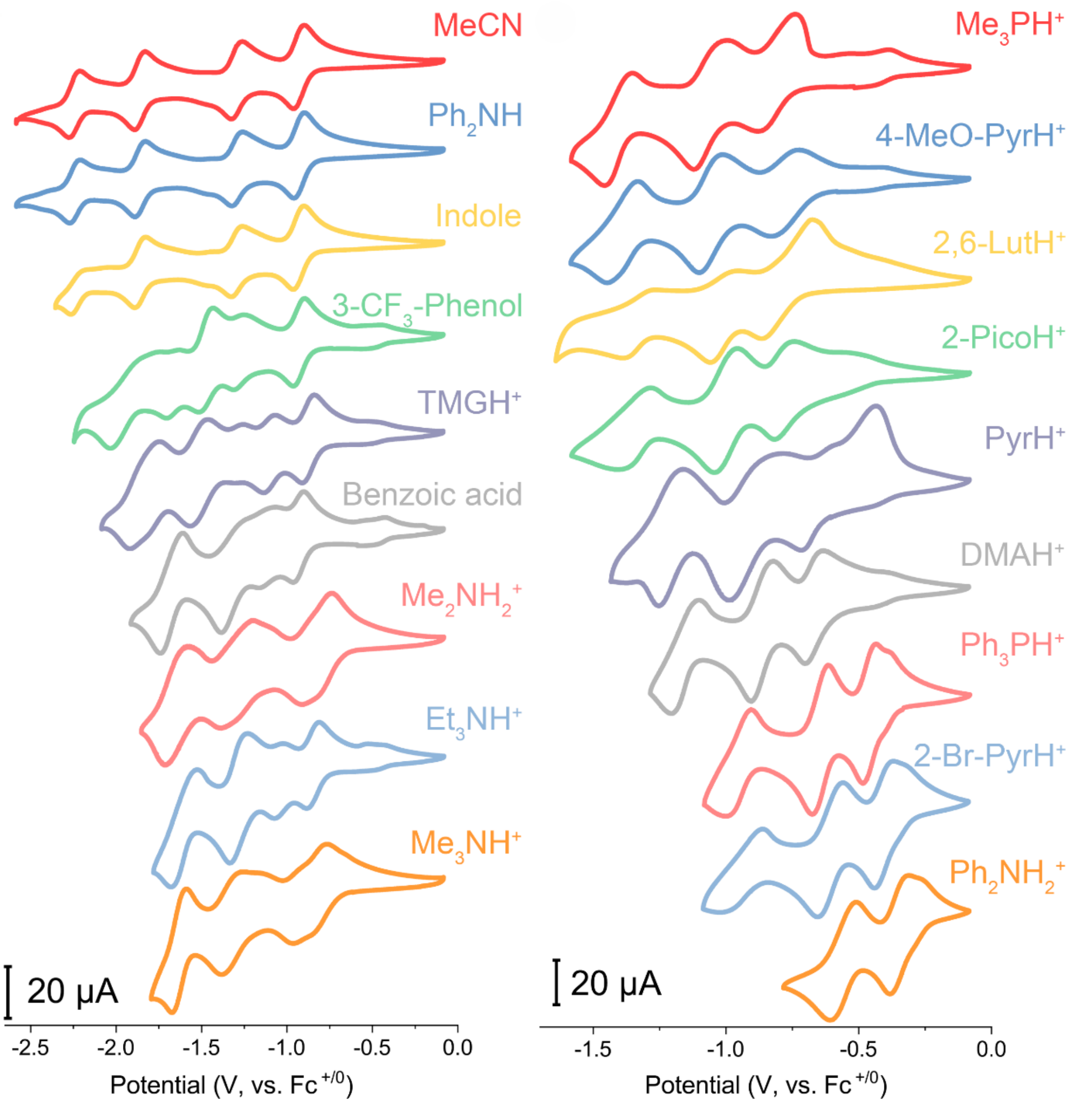

Figure S4. Cyclic voltammograms of 1 mM $\mathbf{P_2W_{18}O_{62}}^{-6}$ obtained in acetonitrile in the presence of 6 mM various organic acids with the scan rate of 100 mV/s, using 0.1 M [^{n}Bu$_4$N]PF$_6$ as the supporting electrolyte. Ferrocene is used for each measurement as the internal standard. The corresponding acids are listed in Table S2.

Table S2. pK_a values of various organic acids in acetonitrile.

Acid	Abbreviation	pK_a(MeCN)	Ref.
Acetonitrile	MeCN	39.5	2, 3
Diphenylamine	Ph_2NH	34.3	2, 4
Indole		32.57	5
3-Trifluoromethyl-Phenol	3-CF_3-phenol	26.5	5
4-Trifluoromethyl-Phenol	4-CF_3-phenol	25.5	5
1,1,3,3-Tetramethylguanidinium tetrafluoroborate	$TMGH^+$	23.35	6
4-Methoxyl-Thiophenol	4-MeO-PhSH		3
Benzoic acid		21.5	7
Piperidinium tetrafluoroborate			6
Dimethyl ammonium tetrafluoroborate	$Me_2NH_2^+$	19.03	6
Triethylammonium chloride	Et_3NH^+	18.83	6
cyclo-Hexylammonium tetrafluoroborate	^{c}HexNH$_3^+$		6
Trimethylammonium chloride	Me_3NH^+	17.61	6
Trimethylphosphonium tetrafluoroborate	Me_3PH^+	15.48	6
4-Methoxyl-Pyridium tetrafluoroborate	4-MeO-PyrH$^+$	14.24	6
2,6-Lutidinium tetrafluoroborate	2,6-LutH$^+$	14.16	6
2-Picodinium tetrafluoroborate	2-PicoH$^+$	13.28	6
Pyridium tetrafluoroborate	PyrH$^+$	12.53	6
N,*N*-Dimethylanilinium tetrafluoroborate	DMAH$^+$	11.47	6
Pyrazinium tetrafluoroborate	Pyrazinium$^+$	7.74	6
Triphenylphosphonium tetrafluoroborate	Ph_3PH^+		6
2-Bromo-pyridium tetrafluoroborate	2-Br-PyrH$^+$	7.02	6
2-Chloro-pyridium tetrafluoroborate	2-Cl-PyrH$^+$	6.79	6
Diphenylammonium tetrafluoroborate	$Ph_2NH_2^+$	5.98	6

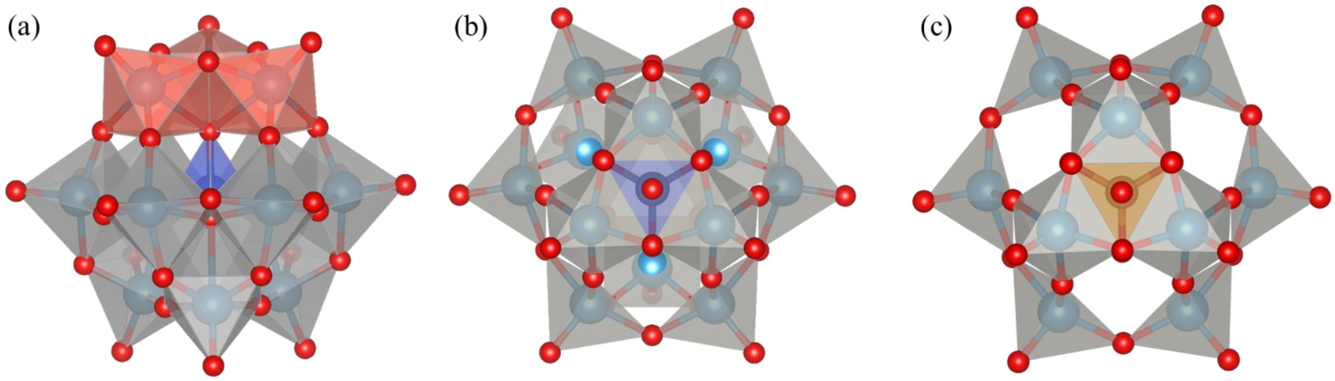

Figure S5. Polyhedra representations of (a) $\mathbf{SiW_{12}O_{40}^{-4}}$ with asymmetric octahedral polyhedra and highlighted 'cap' feature (red octahedra), (b) $\mathbf{SiW_{12}O_{40}^{-4}}$ from the perspective of one of its four C_3 symmetry axes, with square pyramidal polyhedra, and (c) $\mathbf{P_2W_{18}O_{62}^{-6}}$ from the perspective of its C_3 symmetry axis, with square pyramidal polyhedra. Si tetrahedra are colored blue and P tetrahedra are colored orange to distinguish from the surrounding W polyhedra (grey). Belt features comprising the outer W rings are evident in (b) and (c). These structures were geometrically optimized in DFT with their Me_3HN^+ and Me_4N^+ counterions present; for figure clarity the counterions are not shown. 'Edge' and 'corner' features are evident with the asymmetric octahedra in (a). For $\mathbf{SiW_{12}O_{40}^{-4}}$, the edge sites correspond to O_{B1} and the corner sites correspond to O_{B2}. O site characterizations for other clusters are provided in Table S3 and Figure 3 of the main text.

Table S3. List of optimized W–O bond lengths per DFT calculations.

Cluster	Site Type	Location Descriptions		W–O Bond Length (Å)		
				Average	Standard Deviation	% St.Dev.
$\mathbf{W_6O_{19}^{-2}}$	$\mathbf{O_{B1}}$ (120.0°)	Belt / Cap	Edge	1.94	0.00092	0.05%
	$\mathbf{O_{T1}}$	Belt / Cap	Corner	1.73	0.00066	0.04%
	$\mathbf{O_G}$	n/a	n/a	2.38	0.00134	0.06%
$\mathbf{W_{10}O_{32}^{-4}}$	$\mathbf{O_{T2}}$	Cap	Corner	1.75	0.00028	0.02%
	$\mathbf{O_{B3}}$ (119.8°)	Tie	Edge	1.95	0.01457	0.75%
	$\mathbf{O_{B1}}$ (120.0°)	Belt	Edge	1.93	0.00148	0.08%
	$\mathbf{O_{T1}}$	Belt	Corner	1.74	0.00091	0.05%
	$\mathbf{O_{B4}}$ (178.5°)	Seam	Corner	1.91	0.00128	0.07%
	$\mathbf{O_G}$	n/a	n/a	2.36	0.01184	0.50%
$\mathbf{SiW_{12}O_{40}^{-4}}$	$\mathbf{O_{T1}}$	Belt / Cap	Corner	1.74	0.00023	0.01%
	$\mathbf{O_{B1}}$ (124.1°)	Belt / Cap	Edge	1.93	0.00212	0.11%
	$\mathbf{O_{B2}}$ (153.0°)	Belt / Cap	Corner	1.92	0.00446	0.23%
	$\mathbf{O_G}$	n/a	n/a	2.37	0.00167	0.07%
$\mathbf{P_2W_{18}O_{62}^{-6}}$	$\mathbf{O_{T2}}$	Cap	Corner	1.74	0.00071	0.04%
	$\mathbf{O_{B5}}$ (125.0°)	Cap	Edge	1.94	0.00156	0.08%
	$\mathbf{O_{B3}}$ (153.2°)	Tie	Corner	1.93	0.01369	0.71%
	$\mathbf{O_{B1}}$ (125.6°)	Belt	Edge	1.93	0.00121	0.06%
	$\mathbf{O_{B2}}$ (154.2°)	Belt	Corner	1.91	0.00123	0.06%
	$\mathbf{O_{T1}}$	Belt	Corner	1.74	0.00059	0.03%
	$\mathbf{O_{B4}}$ (164.3°)	Seam	Corner	1.91	0.00075	0.04%
	$\mathbf{O_G}$	n/a	n/a	2.37	0.01196	0.51%

The standard deviations for these bond angles are ≤ 0.12°, except for the 153° O_B of $\mathbf{SiW_{12}O_{40}^{-4}}$ that has a standard deviation of 0.55°. In $W_{10}O_{32}^{-4}$ and $P_2W_{18}O_{62}^{-6}$, the O_B–W bond length standard deviations are increased for the thermodynamically favored H-binding sites, with accompanying increased standard deviation of the O_G–W bond lengths.

Table S4. DFT symmetry-breaking energies for each cluster without counterions. Negative values indicate absolute energy relaxation and spontaneity. With appropriate charge in lieu of counterions, symmetrically constrained and unconstrained calculations were performed for each cluster to determine their relative energies. All other geometry optimizations were not symmetry-constrained.

Cluster	Symmetry-Breaking Energy, kcal/mol
$W_6O_{19}^{-2}$	-1.9
$W_{10}O_{32}^{-4}$	-0.3
$SiW_{12}O_{40}^{-4}$	-1.2
$P_2W_{18}O_{62}^{-6}$	-0.9

Table S5. DFT-calculated BDFE(O–H) per 1H incremental reductions for POTs studied in this work.

Cluster	Hydroxide Bond Number $(O\text{–}H)_x$	DFT BDFE(O–H) kcal/mol
$W_6O_{19}^{-2}$	1	35.5
	2	34.9
$W_{10}O_{32}^{-4}$	1	49.5
	2	52.3
	3	35.0
	4	36.2
$SiW_{12}O_{40}^{-4}$	1	46.6
	2	42.7
	3	39.2
	4	37.6
$P_2W_{18}O_{62}^{-6}$	1	50.7
	2	51.0
	3	45.7
	4	46.0
	5	44.4
	6	38.0

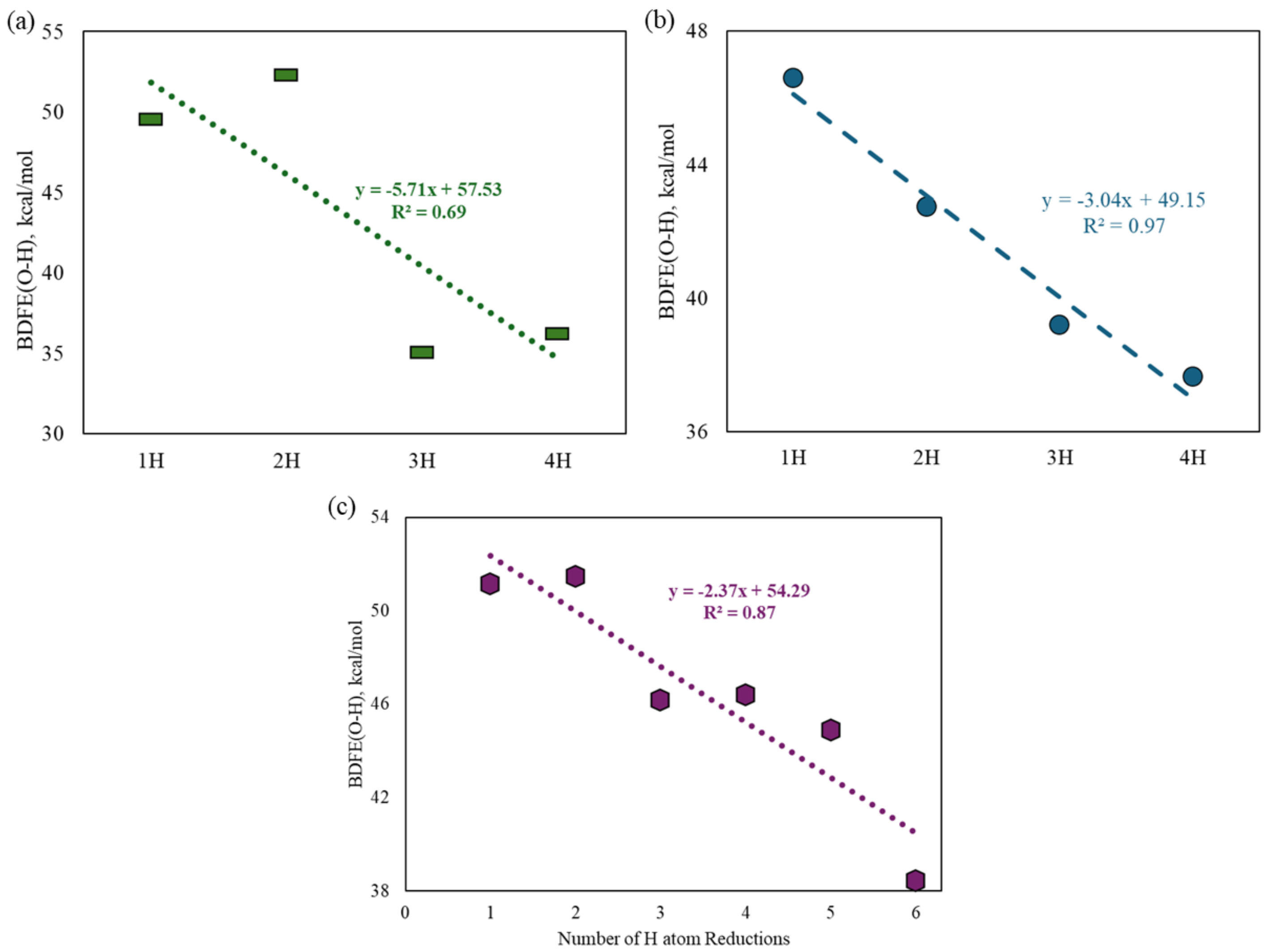

Figure S6. DFT-calculated BDFE(O–H) vs. degree of H reduction for (a) $\mathbf{W_{10}O_{32}}^{-4}$, (b) $\mathbf{SiW_{12}O_{40}}^{-4}$, and (c) $\mathbf{P_2W_{18}O_{62}}^{-6}$. All roughly correspond with experimental values (Table 2 of the main text, Table S5 for individual DFT values, and Table S1 for individual experimental values). Notably, the two ellipsoidal clusters with D symmetries suggest pairs of similar BDFE(O–H), while spherical $\mathbf{SiW_{12}O_{40}}^{-4}$ does not. This seems to suggest that reduction of the spherical clusters impacts the electronic environment of the whole cluster, while single reduction of the D-symmetry ellipsoidal clusters may only impact one reduced side. The two DFT BDFE(O–H) determined for $\mathbf{W_6O_{19}}^{-2}$ were 35.5 and 34.9 kcal/mol, but are not sufficient to suggest trends.

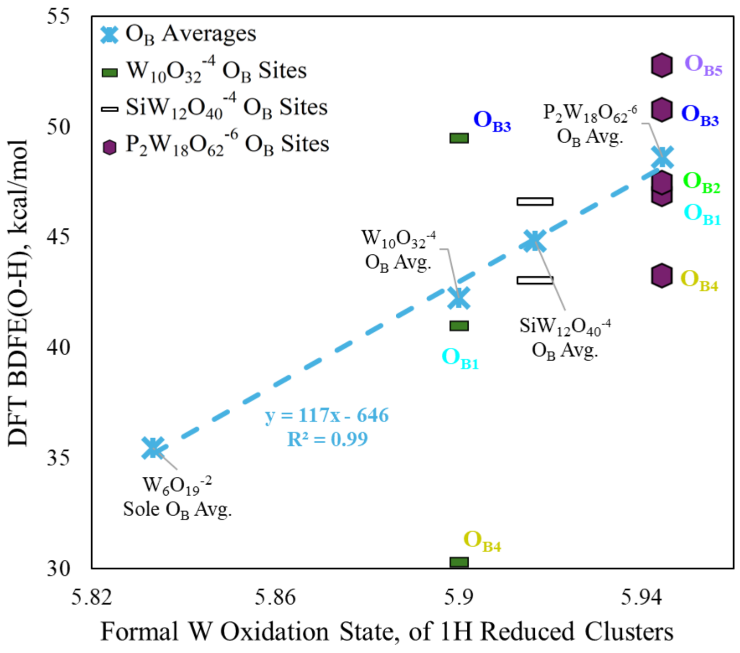

Figure S7. Relationship between DFT-calculated BDFE(O–H) values and formal W oxidation states of 1H reduced POT clusters for different O_B site types (locations as described in main text Figure 3), and their per-cluster site-weighted averages (light blue asterisks). The average formal W oxidation states of the 1H reduced clusters serve as a proxy for cluster size.

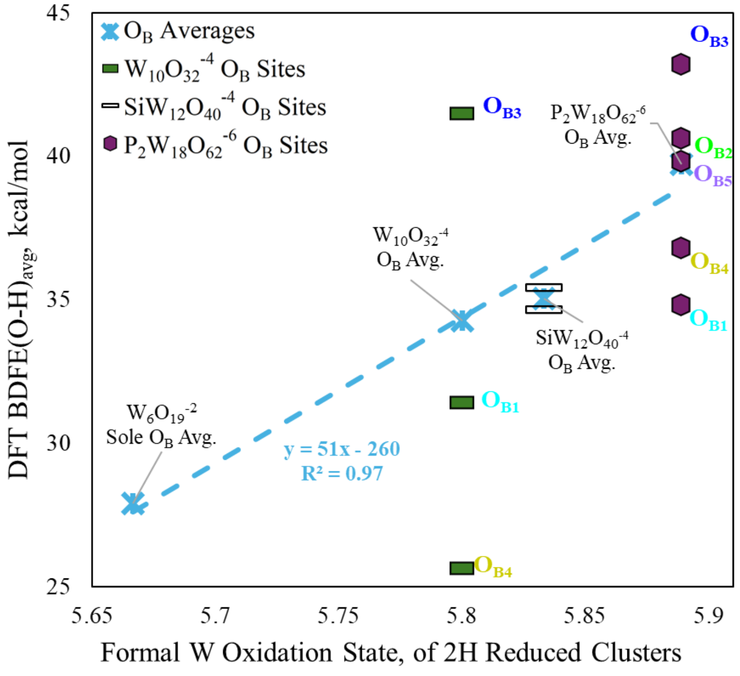

Figure S8. Relationship between DFT BDFE(O–H) values calculated with an alternative def2-TZVP/SVP basis set (see Computational Methods) and formal W oxidation states of 2H reduced POT clusters for different O_B site types (locations as described in main text Figure 3), and their per-cluster site-weighted averages (light blue asterisks). The average formal W oxidation states of the 2H reduced clusters serve as a proxy for cluster size.

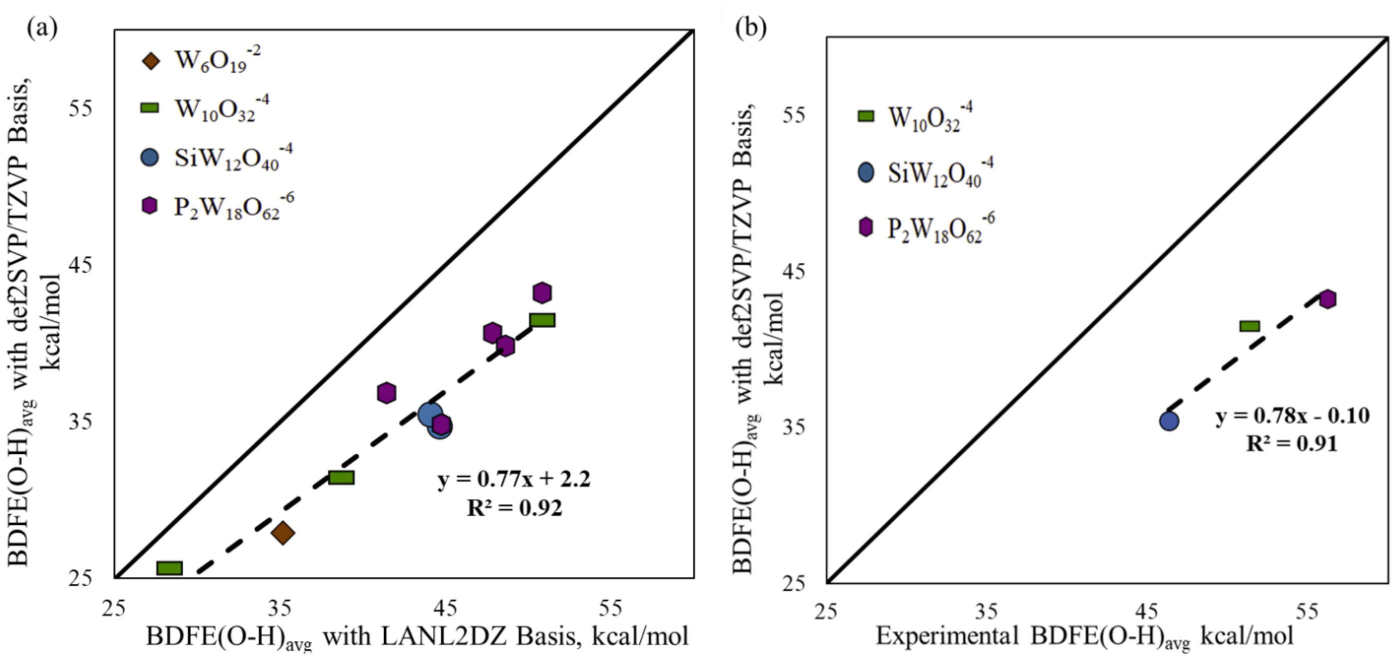

Figure S9. Parity of DFT BDFE(O–H)$_{avg}$ values calculated with an alternative def2-TZVP/SVP basis set (see Computational Methods) against (a) DFT BDFE(O–H)$_{avg}$ calculated with the LANL2DZ basis set, and (b) experimental BDFE(O–H)$_{avg}$ values. The BDFE(O–H)$_{avg}$ values calculated with the alternative basis set deviate from experiments when compared against the LANL2DZ results of main text Figure 4a.

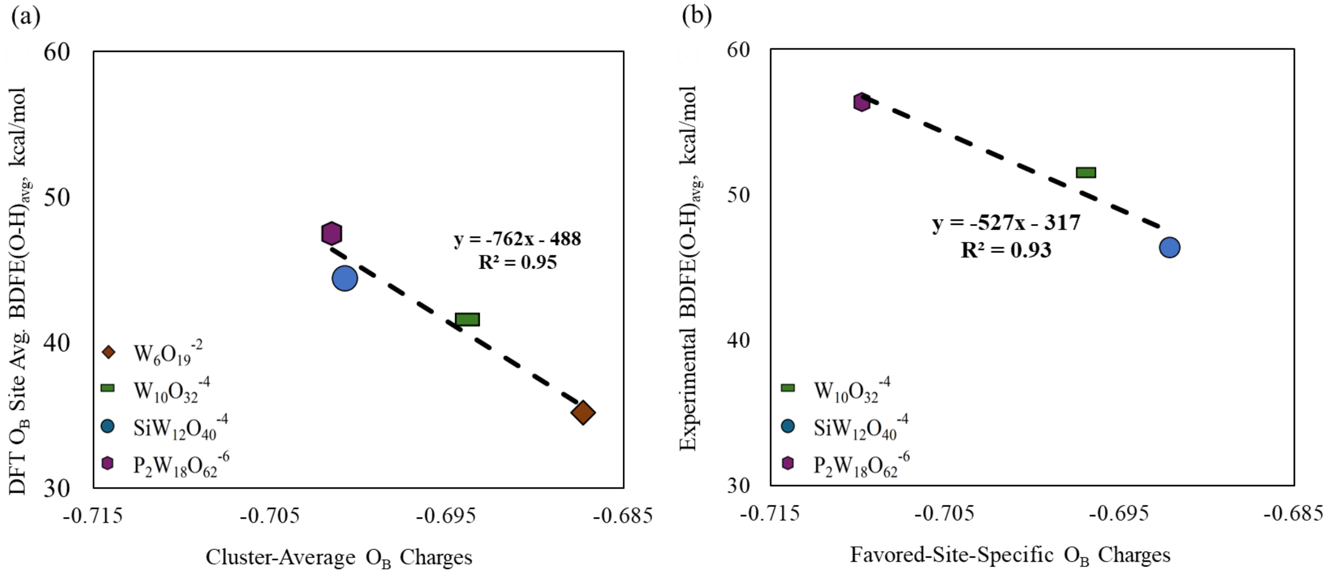

Figure S10. Relation of BDFE(O–H) to unreduced cluster O$_B$ charge for (a) DFT calculated all-O$_B$-site-average BDFE(O–H)$_{avg}$ vs. all-O$_B$-site-average charges, and (b) experimental BDFE(O–H)$_{avg}$ vs. the site-specific O$_B$ charges.

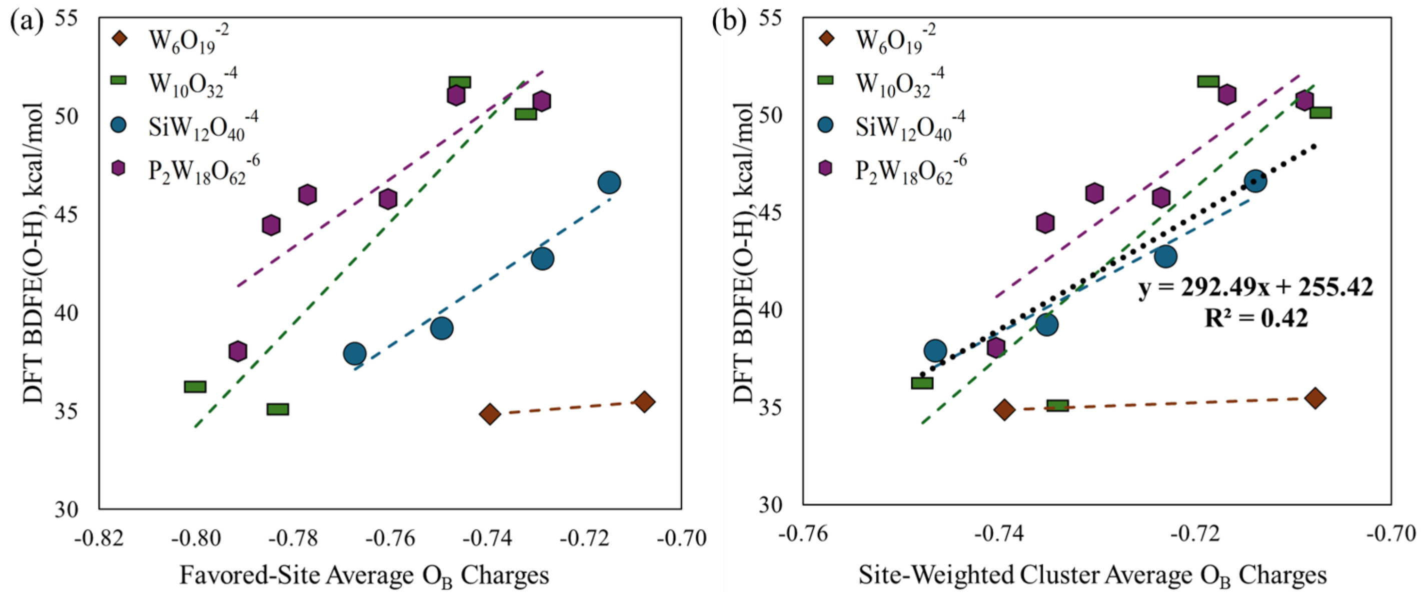

Figure S11. Relation of BDFE(O–H) to (a) thermodynamically favored O_B site-type average charges, and (b) site-weighted cluster-average O_B charges, for clusters with different degrees of H atom reduction, corresponding to those of Figure S6.

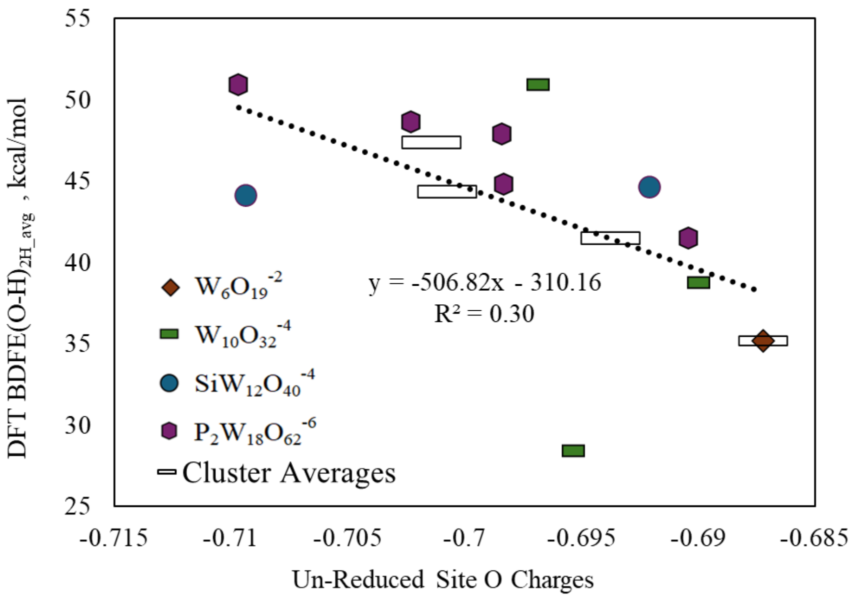

Figure S12. Site-specific and cluster-average DFT BDFE(O–H) vs. site and cluster-average O_B charges (unreduced clusters). The trendline is a best fit of all individual points. The cluster averages are the same as shown in Figure S10a.

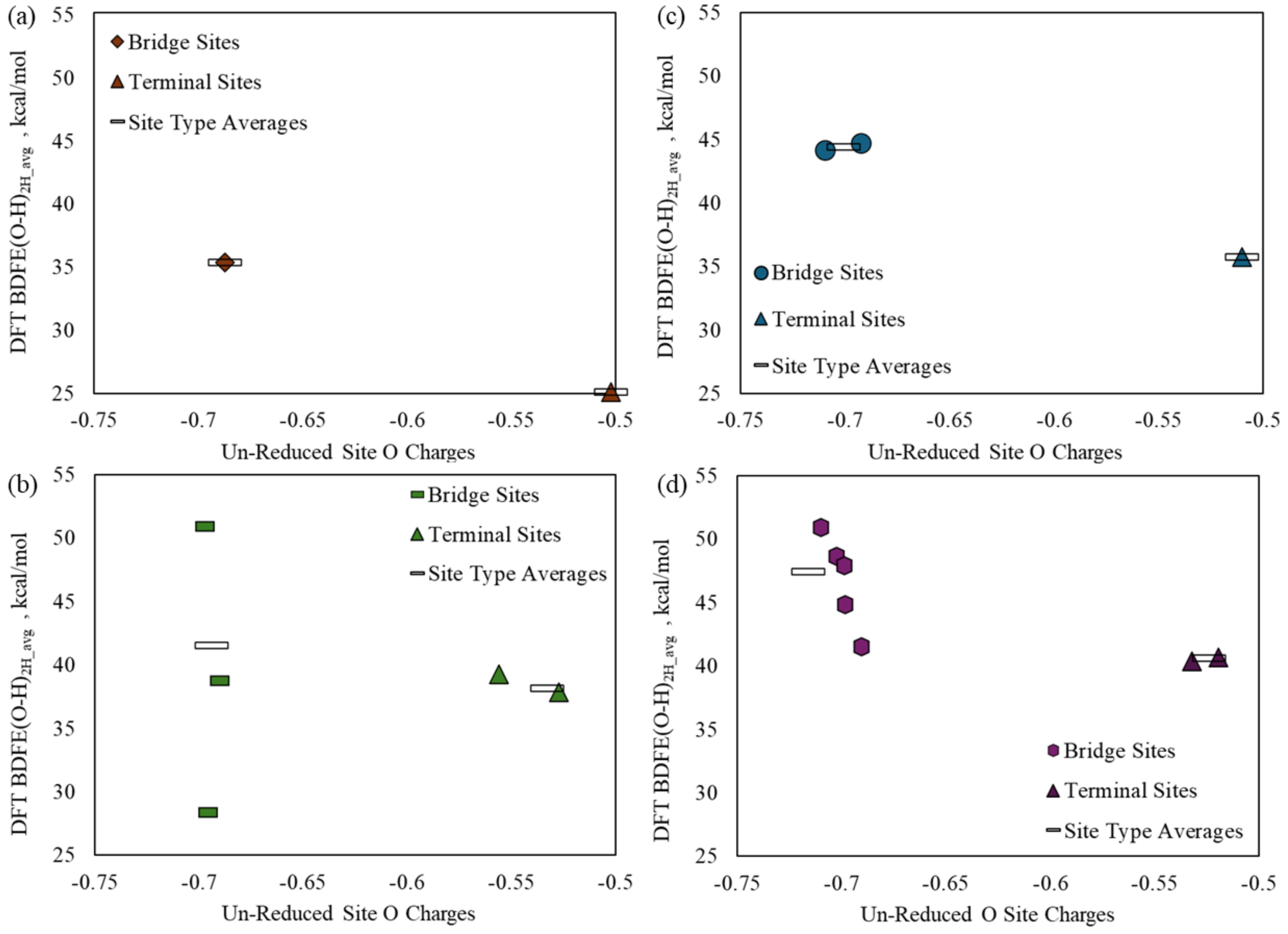

Figure S13. DFT BDFE(O–H) of O_B and O_T sites vs. their site and O_B/O_T-type specific average O charges (unreduced clusters) for (a) $\mathbf{W_6O_{19}^{-2}}$, (b) $\mathbf{W_{10}O_{32}^{-4}}$, (c) $\mathbf{SiW_{12}O_{40}^{-4}}$, and (d) $\mathbf{P_2W_{18}O_{62}^{-6}}$.

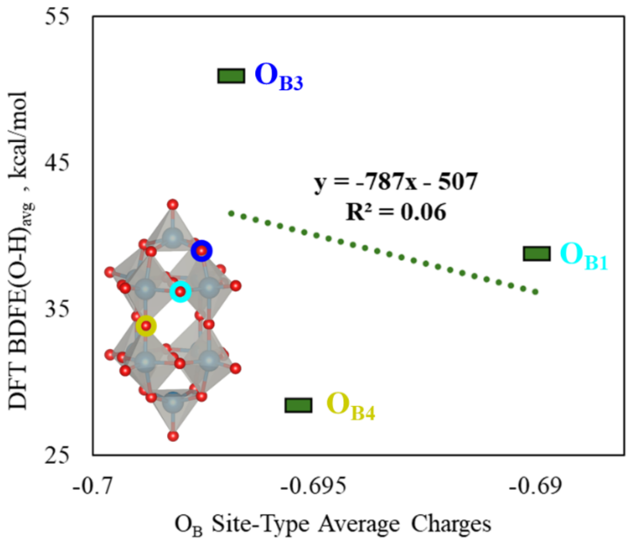

Figure S14. DFT BDFE(O-H)$_{avg}$ vs. site-type specific average O_B charges of unreduced clusters.

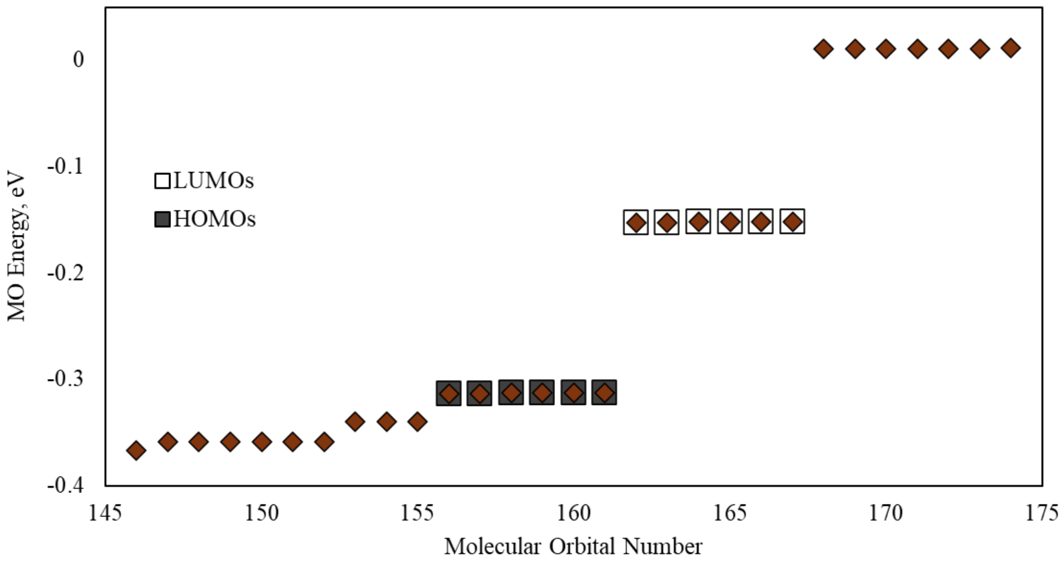

Figure S15. Molecular orbital energies for $\mathbf{W_6O_{19}}^{-2}$, from uB3LYP calculation alpha orbitals, which optimizes to a closed-shell singlet set of equal alpha & beta orbitals. For $\mathbf{W_6O_{19}}^{-2}$ there are 6 essentially isoenergetic HOMOs and LUMOs, corresponding to at least 1 of each per W atom.

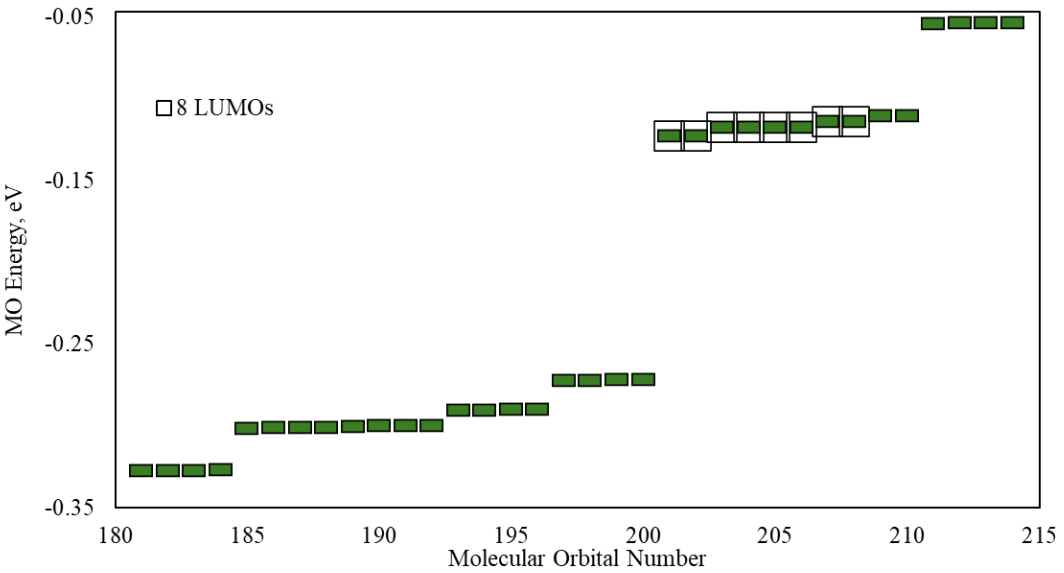

Figure S16. Molecular orbital energies for $\mathbf{W_{10}O_{32}}^{-4}$, from uB3LYP calculation alpha orbitals. For $\mathbf{W_{10}O_{32}}^{-4}$ the two highest-energy LUMOs (the first two without boxes around them) correspond to molecular orbitals around the two “cap” W. In main text Figure 6, the reducing 1e^{-} electron density is delocalized around the belt LUMOs, but does not involve the cap LUMOs. At least 8 occupied frontier HOMOs are also present in relatively similar energetic range to one another.

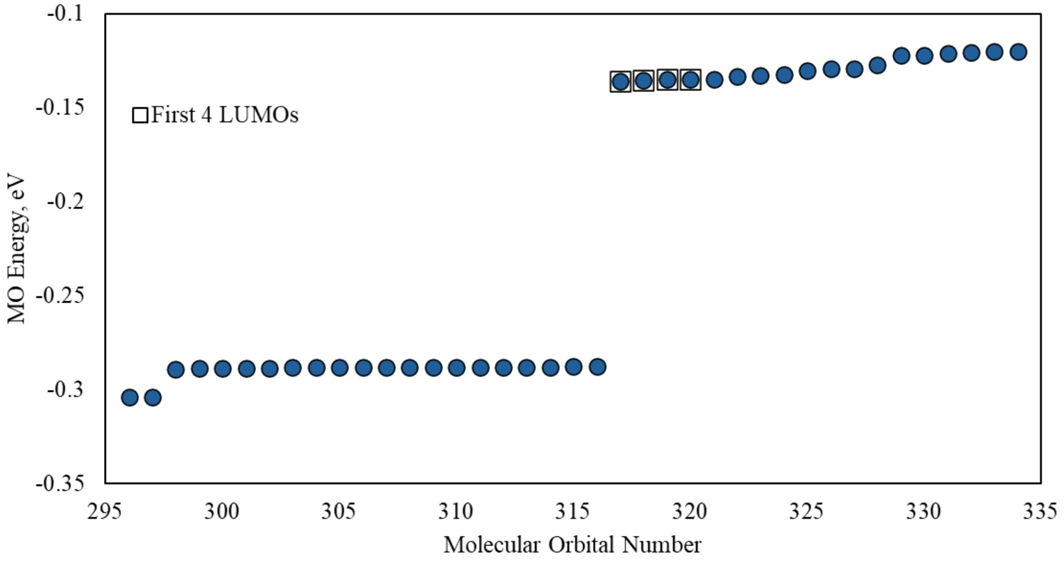

Figure S17. Molecular orbital energies for $\mathbf{SiW_{12}O_{40}^{-4}}$, from uB3LYP calculation alpha orbitals. Frontier occupied and unoccupied molecular orbitals are present in number at least equal to that of the number of W.

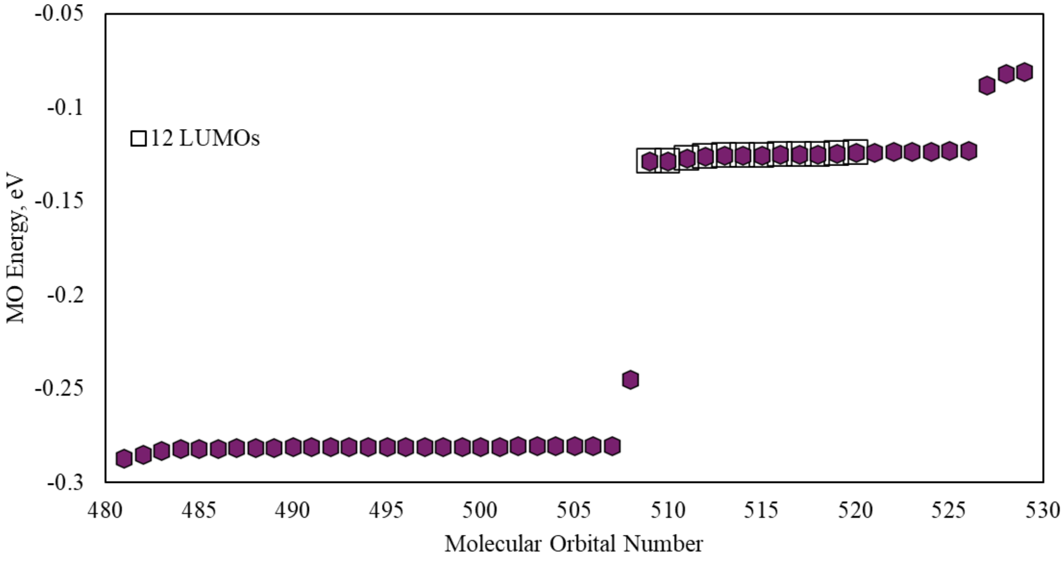

Figure S18. Molecular orbital energies for $\mathbf{P_2W_{18}O_{62}^{-6}}$, from uB3LYP calculation alpha orbitals. Frontier occupied and unoccupied molecular orbitals are present in number at least equal to that of the number of belt W. In main text Figure 6, the reducing 1e^- electron density is delocalized around the belt LUMOs corresponding to 12 W centers.

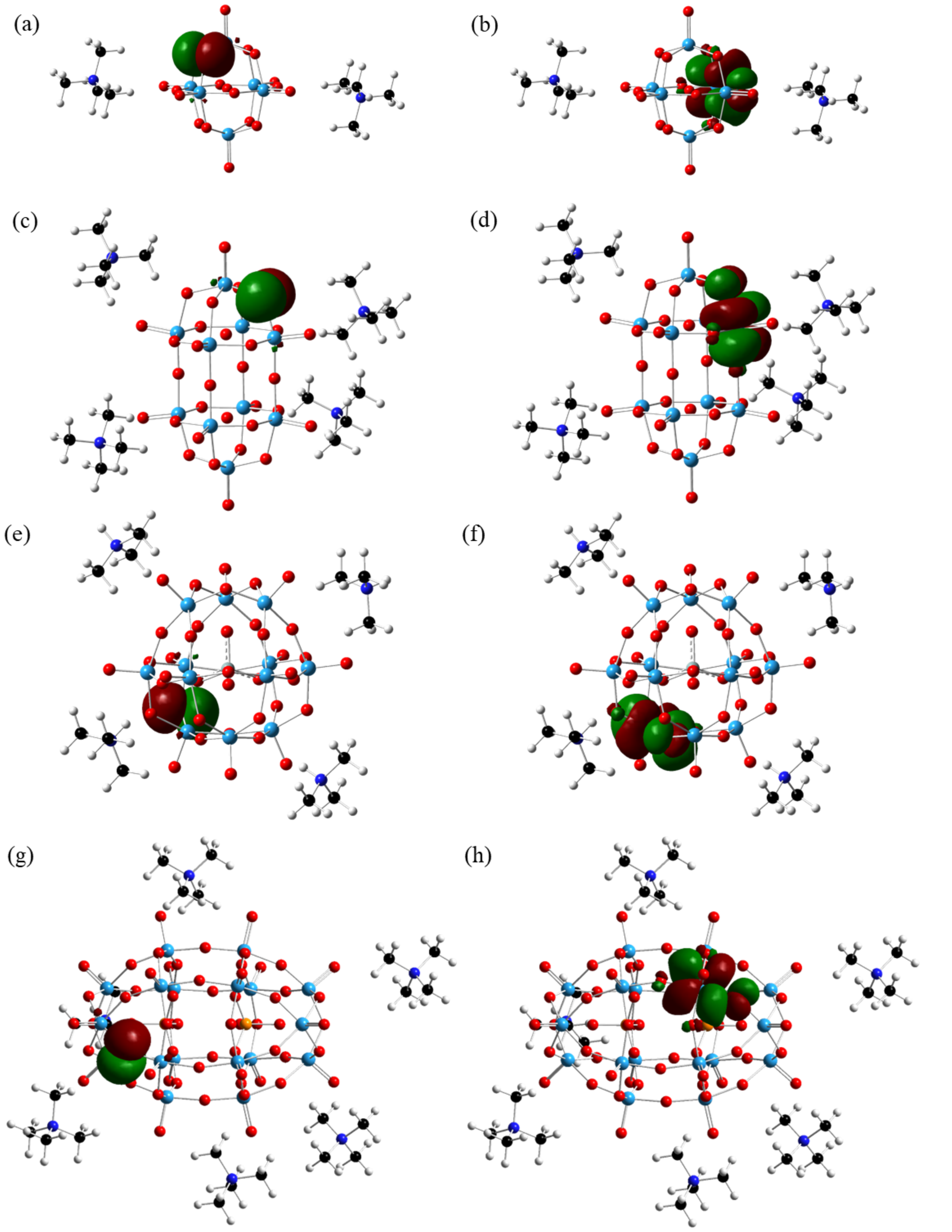

Figure S19. Representative molecular orbitals for (a) $\mathbf{W_6O_{19}^{-2}}$ HOMO, (b) $\mathbf{W_6O_{19}^{-2}}$ LUMO, (c) $\mathbf{W_{10}O_{32}^{-4}}$ HOMO, (d) $\mathbf{W_{10}O_{32}^{-4}}$ LUMO, (e) $\mathbf{SiW_{12}O_{40}^{-4}}$ HOMO, (f) $\mathbf{SiW_{12}O_{40}^{-4}}$ LUMO, (g) $\mathbf{P_2W_{18}O_{62}^{-6}}$ HOMO, and (h) $\mathbf{P_2W_{18}O_{62}^{-6}}$ LUMO. Isosurface density = 0.004.

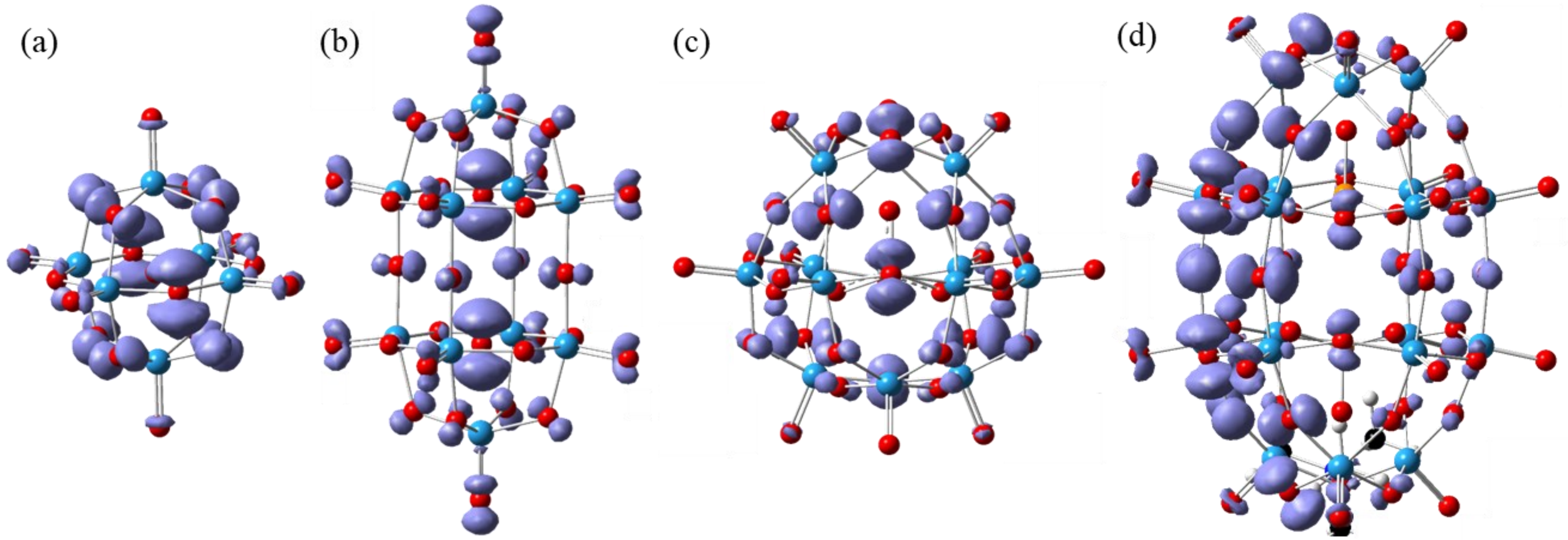

Figure S20. Positive density differences due to 1e^- ionization for (a) $\mathbf{W_6O_{19}^{-2} – W_6O_{19}^{-1}}$, (b) $\mathbf{W_{10}O_{32}^{-4} – W_{10}O_{32}^{-3}}$, (c) $\mathbf{SiW_{12}O_{40}^{-4} – SiW_{12}O_{40}^{-3}}$, and (d) $\mathbf{P_2W_{18}O_{62}^{-6} – P_2W_{18}O_{62}^{-5}}$. Neutral structures were geometrically optimized with counterions and without symmetry restrictions, charged structures were calculated as isostructural electronic single-points from the neutral optimized geometries. Counterions have been removed from the images for clarity, but occupy the same positions illustrated in Figure S1. Isosurface density = 0.002 for $\mathbf{W_6O_{19}^{-2}}$ and $\mathbf{W_{10}O_{32}^{-4}}$; 0.0015 for $\mathbf{SiW_{12}O_{40}^{-4}}$; and 0.001 for $\mathbf{P_2W_{18}O_{62}^{-6}}$.

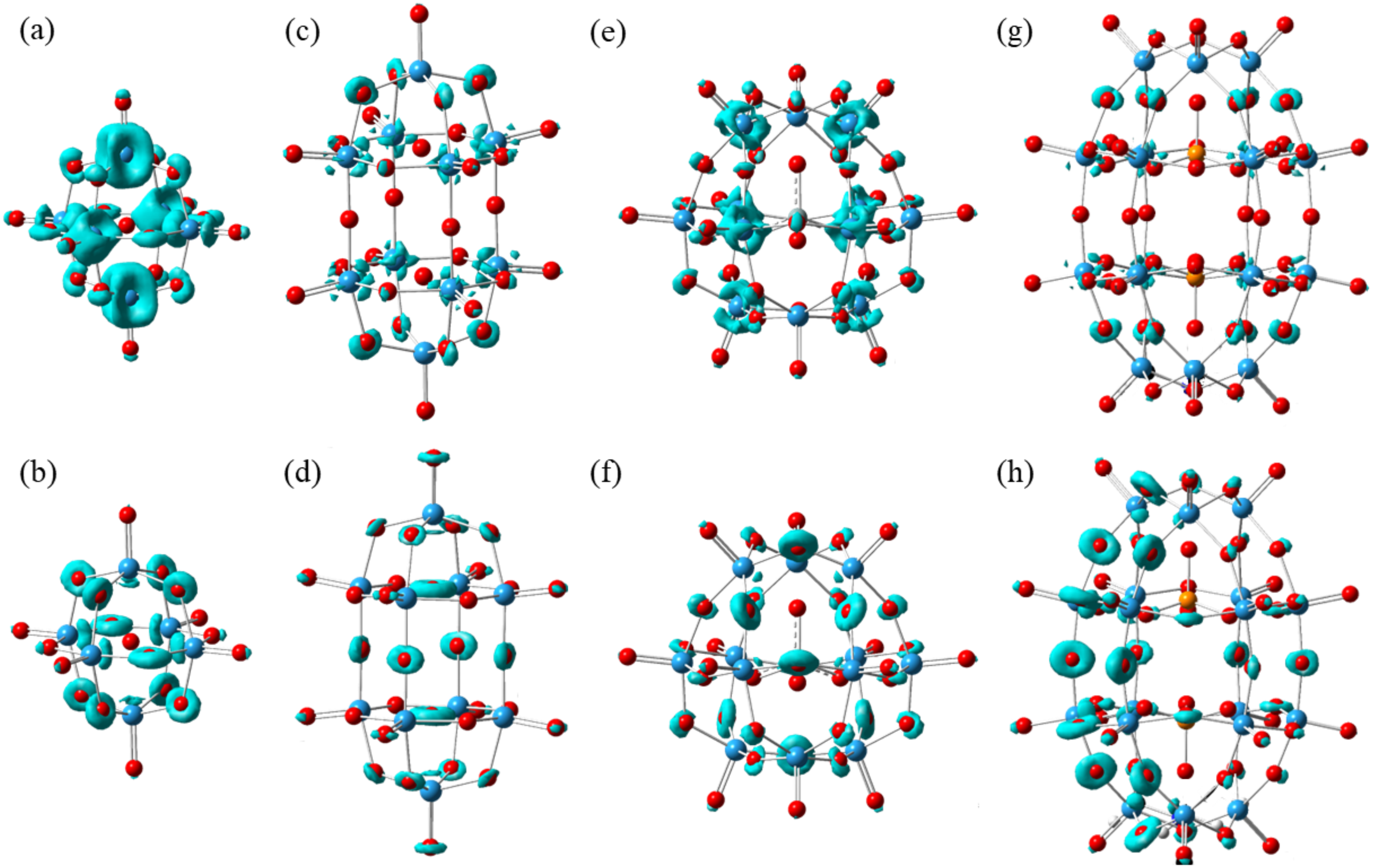

Figure S21. Negative density differences due to 1e^- reduction/ionization for (a) $\mathbf{W_6O_{19}^{-3}}$ – $\mathbf{W_6O_{19}^{-2}}$, (b) $\mathbf{W_6O_{19}^{-2}}$ – $\mathbf{W_6O_{19}^{-1}}$, (c) $\mathbf{W_{10}O_{32}^{-5}}$ – $\mathbf{W_{10}O_{32}^{-4}}$, (d) $\mathbf{W_{10}O_{32}^{-4}}$ – $\mathbf{W_{10}O_{32}^{-3}}$, (e) $\mathbf{SiW_{12}O_{40}^{-5}}$ – $\mathbf{SiW_{12}O_{40}^{-4}}$, (f) $\mathbf{SiW_{12}O_{40}^{-4}}$ – $\mathbf{SiW_{12}O_{40}^{-3}}$, (g) $\mathbf{P_2W_{18}O_{62}^{-7}}$ – $\mathbf{P_2W_{18}O_{62}^{-6}}$, and (h) $\mathbf{P_2W_{18}O_{62}^{-6}}$ – $\mathbf{P_2W_{18}O_{62}^{-5}}$. Neutral structures were geometrically optimized with counterions and without symmetry restrictions, charged structures were calculated as isostructural electronic single-points from the neutral optimized geometries. Counterions have been removed from the images for clarity, but occupy the same positions illustrated in Figure S1. Isosurface density = 0.002 for $\mathbf{W_6O_{19}^{-2}}$ and $\mathbf{W_{10}O_{32}^{-4}}$; 0.0015 for $\mathbf{SiW_{12}O_{40}^{-4}}$; and 0.001 for $\mathbf{P_2W_{18}O_{62}^{-6}}$.

References:

1. Lu, Z.; Dagar, M.; McKone, J. R.; Matson, E. M., Location of dopant dictates proton–coupled electron transfer mechanism in vanadium-substituted polyoxotungstates. *Chem Sci* **2025,** *16* (16), 6736-6743.
2. Yu, H.-Z.; Yang, Y.-M.; Zhang, L.; Dang, Z.-M.; Hu, G.-H., Quantum-Chemical Predictions of pKa's of Thiols in DMSO. *J Phys Chem A* **2014,** *118* (3), 606-622.
3. Bordwell, F. G.; Branca, J. C.; Bares, J. E.; Filler, R., Enhancement of the equilibrium acidities of carbon acids by polyfluoroaryl substituents. *J Org Chem* **1988,** *53* (4), 780-782.
4. Maran, F.; Celadon, D.; Severin, M. G.; Vianello, E., Electrochemical determination of the pKa of weak acids in N,N-dimethylformamide. *J Am Chem Soc* **1991,** *113* (24), 9320-9329.
5. Kütt, A.; Tshepelevitsh, S.; Saame, J.; Lõkov, M.; Kaljurand, I.; Selberg, S.; Leito, I., Strengths of Acids in Acetonitrile. *Eur J Org Chem* **2021,** *2021* (9), 1407-1419.
6. Tshepelevitsh, S.; Kütt, A.; Lõkov, M.; Kaljurand, I.; Saame, J.; Heering, A.; Plieger, P. G.; Vianello, R.; Leito, I., On the Basicity of Organic Bases in Different Media. *Eur J Org Chem* **2019,** *2019* (40), 6735-6748.
7. Vallaro, M.; Ermondi, G.; Saame, J.; Leito, I.; Caron, G., Ionization and lipophilicity in nonpolar media mimicking the cell membrane interior. *Biorg Med Chem* **2023,** *81*, 117203.
